# Macromorphological Control of Zr-Based Metal–Organic Frameworks for Hydrolysis of a Nerve Agent Simulant

**DOI:** 10.1021/acsami.4c11928

**Published:** 2024-09-18

**Authors:** Bradley Gibbons, Eric M. Johnson, Mohammad Khurram Javed, Xiaozhou Yang, Amanda J. Morris

**Affiliations:** Department of Chemistry, Virginia Tech, Blacksburg, Virginia 24061, United States

**Keywords:** metal−organic framework, nerve agent, xerogel, DMNP, hydrolysis, simulant, mesoporosity

## Abstract

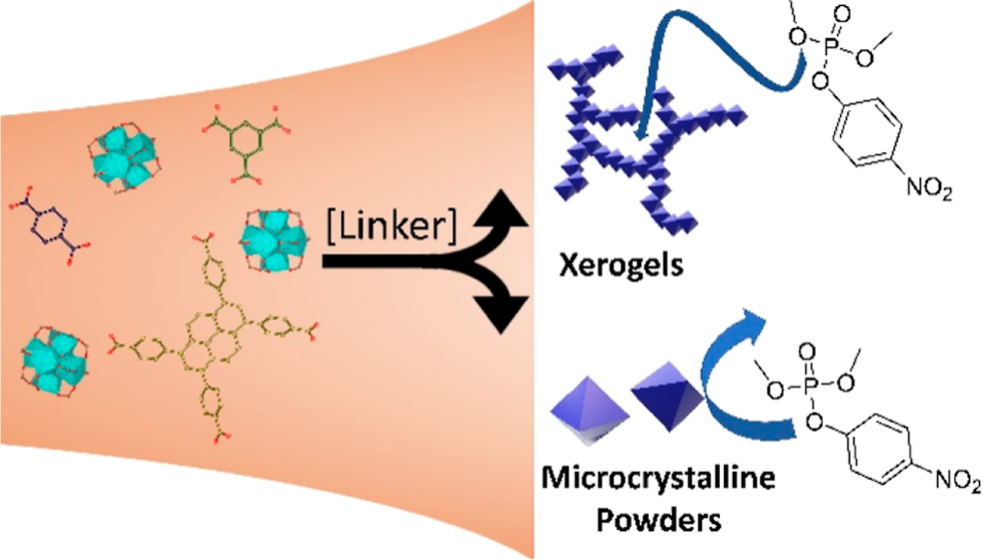

Zirconium-based metal–organic frameworks (MOFs) have become one of the most promising materials for the adsorption and destruction of chemical warfare agents. While numerous studies have shown differences in reactivity based on MOF topology and postsynthetic modification, the understanding of how modifying MOF macromorphology is less understood. MOF xerogels demonstrate modified defect levels and larger porosity, which increase the number of and access to potential active sites. Indeed, UiO-66 and NU-901 xerogels display reaction rates 2 and 3 times higher, respectively, for the hydrolysis of DMNP relative to their powder morphologies. Upon recycling, MOF-808 xerogel outperforms MOF-808 powder, previously noted as the fastest Zr_6_ MOF for hydrolysis of organophosphate nerve agents. The increase in reactivity is largely driven by a higher external surface area and the introduction of mesoporosity to previously microporous materials.

## 1 Introduction

Chemical warfare agents (CWAs) represent a major threat to military personnel and civilians alike due to their high lethality.^1−5^ Rapid detoxification of CWAs is critical to protect large populations.^5−14^ Of particular concern are nerve agents, a class of CWAs that inhibit the breakdown of acetylcholine. The organophosphorus moiety present in most nerve agents forms a strong covalent bond with acetylcholinesterase, causing acetylcholine to continuously tell muscles and other organs to contract.^15,16^ Modern methods of protection rely largely on impregnated active carbons; however, these carbons are more catchalls, which can suffer from slow degradation kinetics and limited uptake.^5,14^ The development of materials designed with specific interactions with CWAs that can more effectively uptake and decompose nerve agents is needed for enhanced protection from these dangerous chemicals.

Metal–organic frameworks (MOFs) consist of metal nodes linked through organic compounds in highly crystalline networks. The high surface areas, permanent porosity, and high tunability of MOFs make this class of materials a promising candidate for the uptake and sequestration of nerve agents. Many MOFs have been screened for reactivity toward nerve agents and simulants, with Zn-^17−19^ and Zr-based^20−30^ MOFs as standouts. The Zn–O–Zn and Zr–O–Zr node structures in these MOFs mimic phosphotriesterase, an enzyme known to destroy organophosphorus-based compounds including nerve agents.^12,31,32^

Zr-based MOFs are of particular interest due to the large number of MOFs sharing the basic Zr node structure. By change of the coordination number, shape, and size of the organic linker, different topologies and pore sizes emerge from the otherwise identical node. MOFs of note include UiO-66, MOF-808, and NU-901, which all share a Zr_6_O_8_ node but utilize di-, tri-, and tetracarboxylic acid-containing linkers, respectively. From a fundamental perspective, this allows for a direct measure of reactivity as a function of the 3D structure. More practically, the linker differences lead to undercoordination in MOF-808 along with larger pore diameters, which result in enhanced uptake of agents and increased access to active sites.^28,33,34^

One underexplored aspect of MOF-based catalysis is the range of material properties that can be achieved within the same framework due to different macromorphologies (i.e., gel, glass, etc.) that are attainable by modifying reaction conditions.^35^ Although harder to characterize, these materials offer properties not obtainable in MOF powders, such as a higher external surface area and the introduction of mesoporosity into typical microporous structures. These properties are reported to play a significant role in catalytic rate and uptake.^36−40^ As we reach the limits of what we can accomplish with microcrystalline powders, other methods of synthesis are necessary to further probe the structure–function relationships.

In this work, we examined the differences between the MOF xerogels and powders of three Zr MOFs: UiO-66, MOF-808, and NU-901. By increasing the linker: Zr ratio, the morphology of the MOF was shifted from powder to gel form. Direct activation of the wet gel resulted in a MOF xerogel with significantly enhanced mesoporosity compared to that of the MOF powders. The MOFs were tested as hydrolysis catalysts for the decomposition of the G-series nerve-agent simulant dimethyl(4-nitrophenyl)phosphate (DMNP).

## 2 Results and Discussion

**Figure 1:**
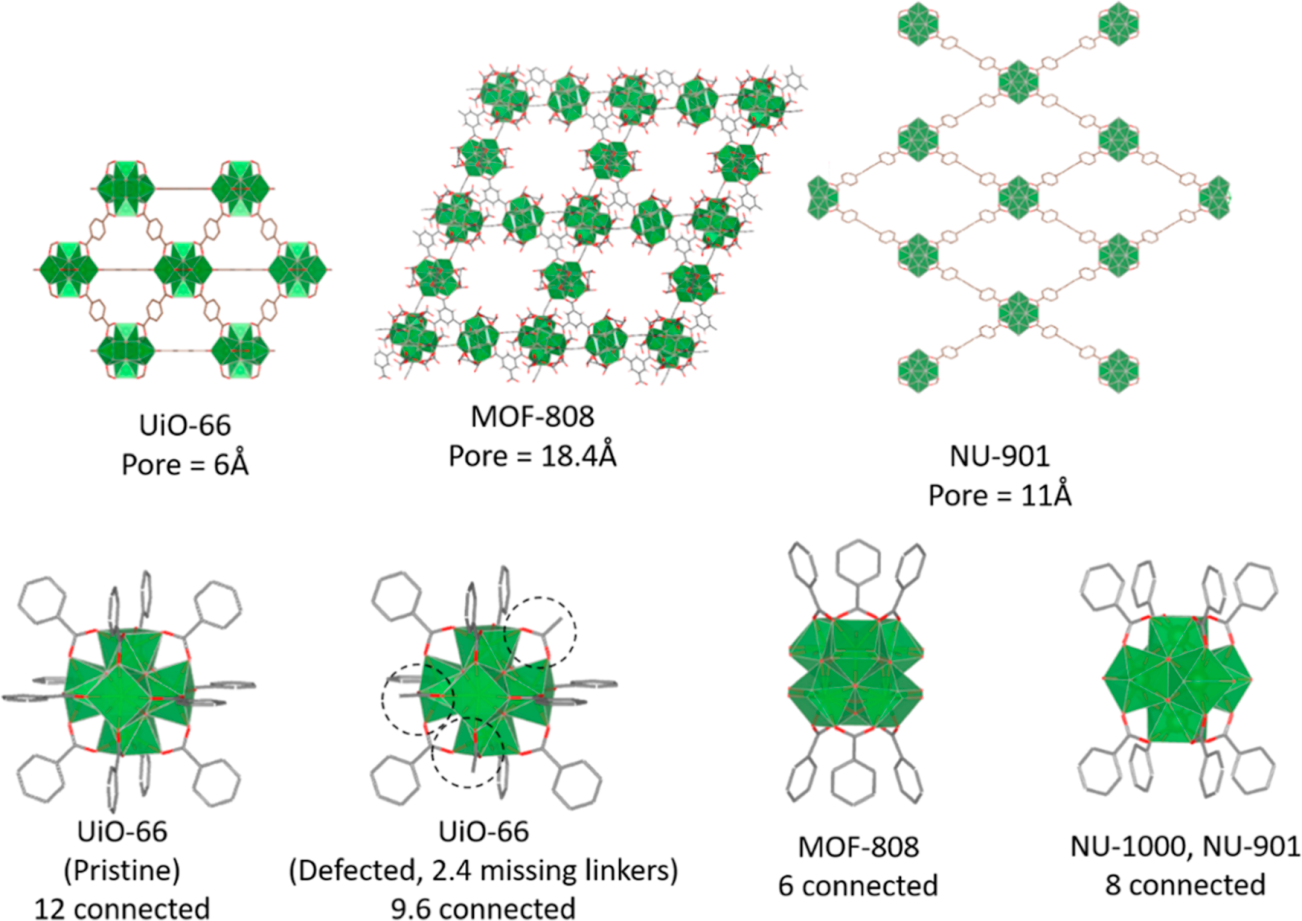
(Top) MOF structures for UiO-66, MOF-808, and NU-901, and (bottom) node structures for UiO-66 (pristine and defected), MOF-808, and NU-901.

Gels^40^ and powders^36,41,42^ of UiO-66, MOF-808, and NU-901 (powder) (Figure 1) were synthesized following literature procedures (see Supporting Information for details). As previously demonstrated, the linker/metal ratio is the critical factor that determines macromorphology, with higher ratios producing gels.^40^ The gels were isolated as wet, nonflowing solids. Drying the MOF gels from ethanol at 200 °C for 2 h produced the respective MOF xerogels.

**Figure 2:**
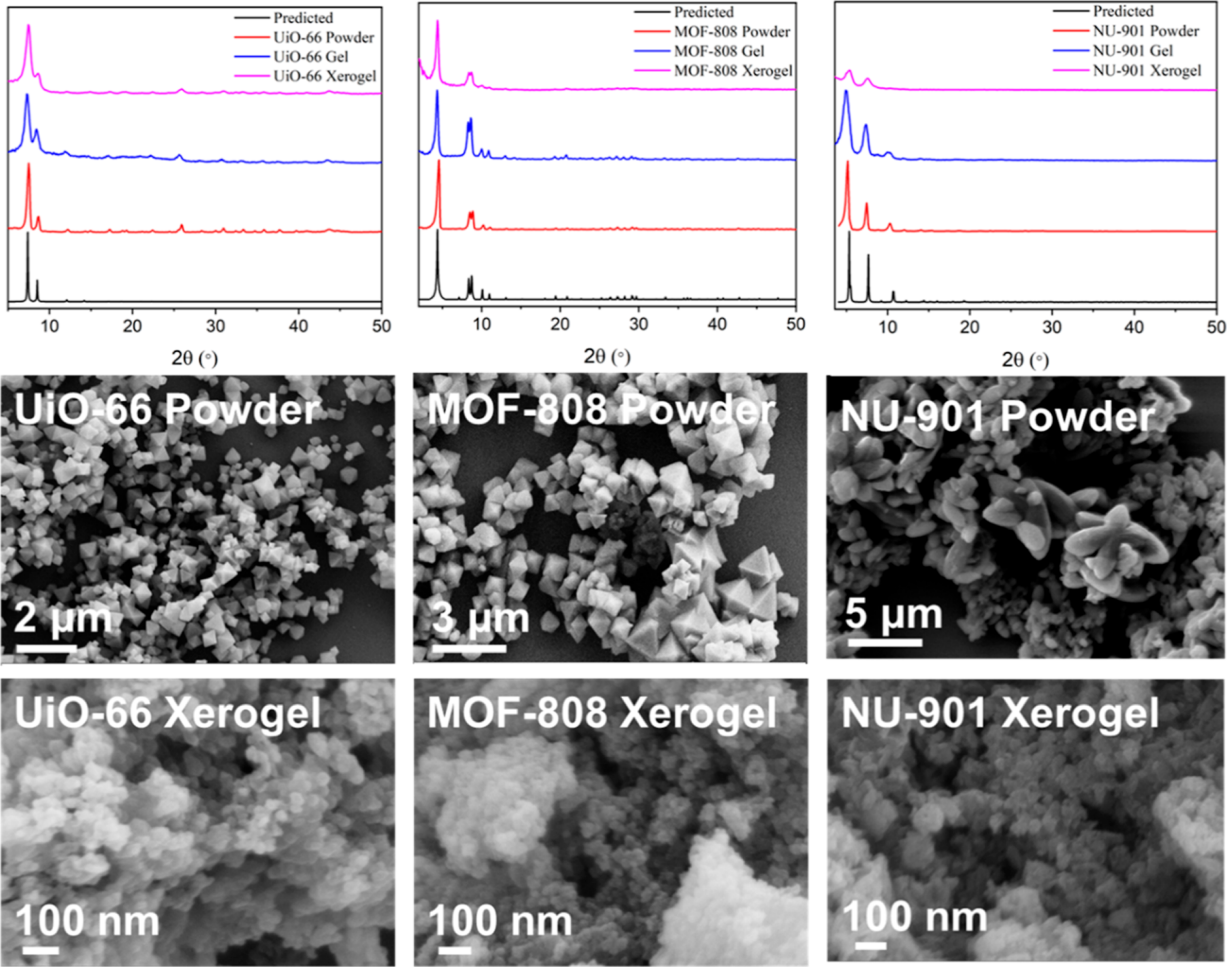
PXRD of UiO-66, MOF-808, and NU-901 powders, gels, and xerogels (top), SEM of UiO-66, MOF-808, and NU-901 powders (middle), and SEM of UiO-66, MOF-808, and NU-901 xerogels (bottom).

Powder X-ray diffraction (PXRD) was conducted on both the MOF gels and xerogels (Figure 2). In all cases, the experimental PXRD patterns matched the predicted patterns for each MOF. The gels displayed broad features at higher angles due to the incorporation of disordered solvent in and around the MOF particles, which was eliminated after drying at high temperatures.^40^ The peaks in the gel and xerogel were also broader (larger width at half-maximum) than the same peaks in the powder sample. A crystallite size (D) was calculated from the Scherrer equation (eq 1) for the MOF powders and xerogels
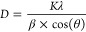
1where *K* is the Scherrer constant (approximated to be 0.89 for all samples), λ is the wavelength of the X-ray source (nm), β is the full width at half-maximum for a peak (rad), and θ is the angle of peak max (rad). To get an average, multiple peaks were used, and the average crystallite size for each sample is reported in Table 1. While not identical to particle size, which is measured by scanning electron microscopy (SEM, vide infra), the crystallite size as calculated by the Scherrer equation is proportional to average particle size. For each structure, the xerogel crystallite size was smaller, approximately half of the size for the powder samples, resulting in an increase in grain boundaries for these samples.^37^

**Table 1:** Crystallite Size, Particle Size, and BET Surface Area for MOF Powders and Xerogels

sample	crystallite size (nm)	particle size (nm)	BET S.A. (m^2^/g)
UiO-66 powder	18 ± 6	700	1402
UiO-66 xerogel	10 ± 2	35	1513
MOF-808 powder	22 ± 7	900	1652
MOF-808 xerogel	10 ± 4	25	450
NU-901 powder	19 ± 2	518	1777
NU-901 xerogel	6 ± 2	45	1844

The size of the MOF particles was determined by using SEM (Figure 2). The powders of UiO-66, MOF-808, and NU-901 have an octahedral morphology, with particle sizes roughly 700, 900, and 518 nm, respectively. The xerogels were all significantly smaller in size, with particle lengths <50 nm in all cases (UiO-66 = 35 nm, MOF-808 = 25 nm, and NU-901 = 45 nm). The UiO-66 and MOF-808 xerogels lack the well-defined shape of the powder samples, instead appearing as closely packed nanoparticles as seen in the literature.^5,40^ Some rod-shaped particles can be seen in the NU-901 xerogel (Figure S5), but overall, the particles are far less defined than in the powder sample. Additionally, the packing of the MOF nanoparticles in the xerogel samples appears to have developed pockets, which may be indicative of mesopores.

**Figure 3:**
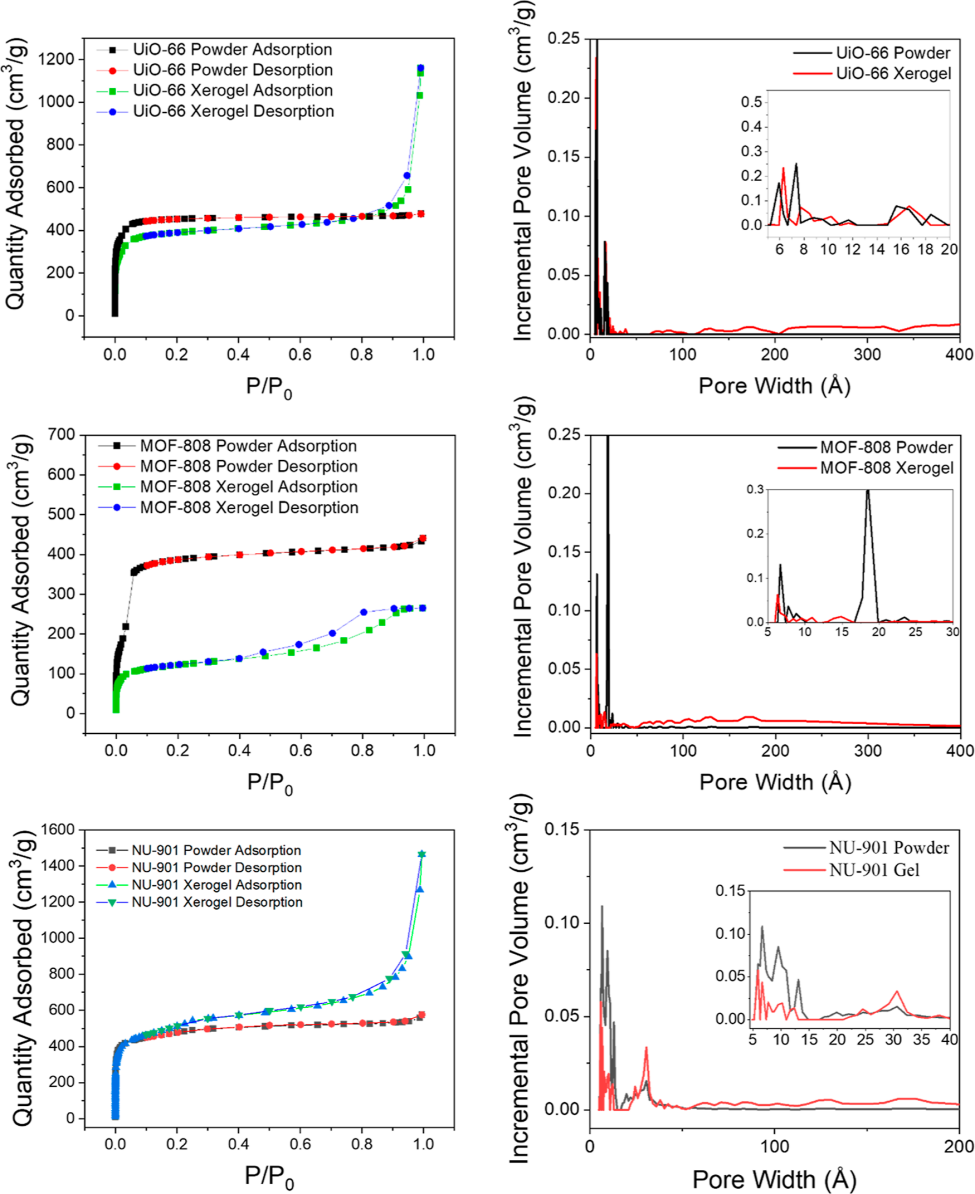
Nitrogen adsorption/desorption isotherms (left) and calculated pore size distribution plots (right) for UiO-66 (top), MOF-808 (middle), and NU-901 (bottom). MOF powder is shown in black/red in the adsorption isotherms and black in the pore size distribution plot, and MOF xerogel is shown in green/blue in isotherms and red in the pore size distribution plot.

Nitrogen adsorption isotherms for the MOF xerogels compared to powders are presented in Figure 3. The BET surface area was calculated by fitting the low P/P_0_ region to a linear BET plot, and the calculated surface areas are summarized in Tables 1 and S1. The adsorption isotherm for both the UiO-66 and NU-901 xerogels resembled type II isotherms and displayed an uptick at high P/P_0_. This uptick could be attributed to either the presence of mesopores within the material or condensation on the external surface. This represents a shift from the traditional type I isotherm observed for UiO-66 and NU-901, respectively.^42^ For NU-901, this shift has isotherm behavior and is further support of the formation of NU-901 xerogel rather than NU-901 powder. While pore size distribution plots showed significant changes between the powders and xerogels, the characteristic pore sizes for each framework were still present in the xerogels, although at a lower intensity due to the presence of mesopores ranging in size from 50 to 400 Å. This, combined with the PXRD results, does not suggest a loss of pore structure but rather the addition of a new void space located between the primary MOF particles and condensation on external surfaces. Unlike the UiO-66 and NU-901 xerogels, the MOF-808 xerogel displayed a lower overall BET surface area compared to the powder, despite evidence of new mesopore formation. The loop present in the desorption isotherm for MOF-808 xerogel is typically observed in activated silica gels and indicative of small pore apertures, leading to pore-blocking and inaccessibility of large mesopores.^43−45^ It is not fully clear why the MOF-808 xerogel displayed significantly lower porosity, although other literature reports have reported similar findings.^46^ Some pore-blocking may result from excess linker connected to the Zr_6_ node, which can be observed by ^1^H NMR and thermogravimetric analysis (TGA) experiments (vide infra). Additionally, the xerogel formation process itself might induce structural changes, such as partial pore collapse or altered interparticle interactions, which further contribute to the reduced porosity. These combined factors suggest that the unique chemical environment of the MOF-808 xerogel may inherently limit its surface area, distinguishing it from other MOF xerogels that exhibit increased porosity compared to their powdered counterparts.

**Figure 4:**
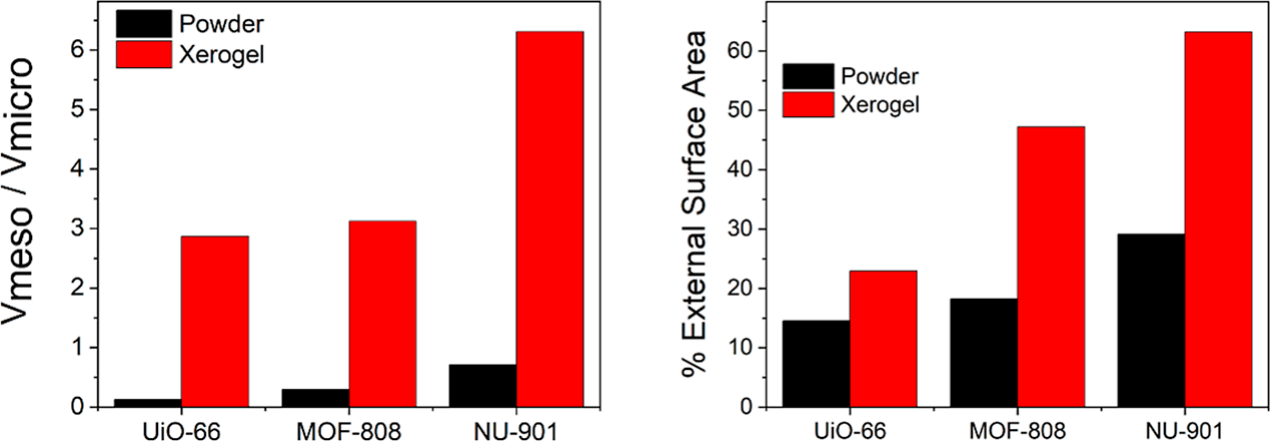
Mesoporosity and external surface area comparison between MOF powder and xerogel. (Left) Ratio of mesopore to micropore volume, with significant enhancement in mesoporosity in MOF xerogels compared to powder analogues. Ratio was calculated and the *t* = pot micropore volume (V_micro_). (Right) Calculated external surface area percentage over total surface area, with significant increase in external surface area in MOF xerogels.

The MOF xerogels all showed significant microporosity in pore size distribution analyses, along with mesopores ranging from 50 to 400 Å in diameter. The total mesopore volume for each sample was calculated by using the BJH adsorption cumulative volume between 17 and 3000 Å pores (V_meso_). A micropore volume (V_micro_) was calculated by the t-plot method, where the thickness of an adsorbate is plotted compared to the adsorption capacity. The ratio of V_meso_ to the t-plot micropore volume (V_micro_) was calculated and plotted in Figure 4, which demonstrated a significant increase in the ratio of mesopore to micropore volume in the MOF xerogels comparable to the powders. Additionally, the external surface area was estimated from the slope of the t-plot and divided by the BET surface area to calculate a percent external surface aera for each sample (Figure 4, right).^47^ MOF xerogels exhibited significant enhancements in mesopore volume and external surface area compared with the MOF powders, suggesting a large network of interparticle mesopores connecting nanosized MOF particles.

**Figure 5:**
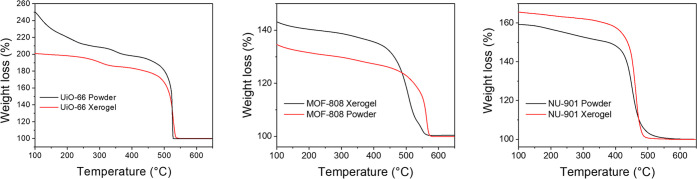
TGA plots for all MOF powders (black) and gels (red), run under air. Left: UiO-66, middle: MOF-808, and right: NU-901.

To obtain larger pores in typically microporous frameworks, purposely promoting missing linkers or nodes (termed defects) is often necessary. TGA has been a standard characterization technique for MOFs to determine the average amount of organic linker present on each node, often termed missing linker defects. Although mostly popular in studies of missing linker defects in UiO-66,^36,48^ the technique can be applied to any Zr-based MOF. In general, each framework displayed similar weight loss behavior, although the magnitude and onset temperature shifted, likely due to different pore geometries and thermal stability of each linker. UiO-66 (Figures 5, left, and S6) has three discrete plateaus, with the first occurring between 100 and 250 °C, the second starting about 350 °C, and the final occurring at 600 °C. Weight loss at temperatures below 100 °C is due to the removal of adsorbed water molecules or volatile organic molecules such as acetone or ethanol, which are commonly used in MOF washing procedures. The next weight loss is associated with the removal of water, hydroxyls, and other capping groups, such as monocarboxylate ligands from the Zr_6_ nodes, resulting in a generalized MOF formula that can be expressed as Zr_6_O_8_(linker)_*x*_, where *X* depends on the specific framework and defect level of the specific sample. The final weight loss is the removal of the structural organic linkers, resulting in only ZrO_2_ remaining at ∼650 °C. By comparison of the weight loss due to organic linkers in any given sample to the ideal case, an average number of linkers per node can be calculated for each sample.

In the case of UiO-66, the xerogel is slightly more defected, with an average of 4.25 linkers per node (Zr_6_O_8_(BDC)_4.25_) compared to the 4.9 linkers per node (Zr_6_O_8_(BDC)_4.9_) in the powder sample. Both samples are defected relative to that of pristine UiO-66 (Zr_6_O_8_(BDC)_6_). A similar trend is present for the NU-901 powder and NU-901 xerogel as well, with the xerogel being highly defected (Zr_6_O_8_(TBAPy)_0.65_). Unlike UiO-66 and NU-901 xerogels, the MOF-808 xerogel showed a much higher weight loss than the powder sample, which indicates a larger amount of organic linker in the xerogel than the powder (Figure 5, middle). The number of linkers calculated for MOF-808 at the onset of linker decomposition (450 °C) reveals a pristine dehydroxylated MOF-808 powder (Zr_6_O_8_(1,3,5-BTC)_2_). The higher weight percent for the MOF-808 xerogel therefore likely corresponds to excess trimesic acid linker in the MOF, giving the formula Zr_6_O_8_(1,3,5-BTC)_4.64_. In general, MOF-808 displays less well-resolved plateaus to other Zr-MOFs such as UiO-66, making it difficult to identify the exact nature of the additional components.^49^ Additional weight at low temperatures suggests incorporation of more adsorbed water or solvent molecules, likely due to the increased porosity (vide infra).

To confirm the TGA predicted molecular formulas for each MOF, the xerogels were digested in basic D_2_O and analyzed by ^1^H NMR (Figures S7–S9). The linker, modulator(s), and trapped solvents were measured against an internal standard of 2,2,2-trifluoroethanol in order to quantify each molecule present. NU-901 and UiO-66 xerogels showed good agreement between the TGA calculations and ^1^H NMR calculations, confirming that the xerogels are indeed more defective than the powders. The MOF-808 xerogel, in contrast, displayed an equimolar amount of acetone trapped in the pores to 1,3,5-BTC in the MOF. Even with acetone trapped in the pores, the MOF-808 xerogel still demonstrated higher than expected linkers per node, with a formula unit of Zr_6_O_8_(BTC)_3.45_. MOF-808 is typically highly undercoordinated, with 6 open metal sites per node, but the high concentration of linker present in xerogel synthesis has likely led to BTC capping agents on MOF-808 nodes, giving rise to a more coordinatively saturated node.

**Figure 6:**
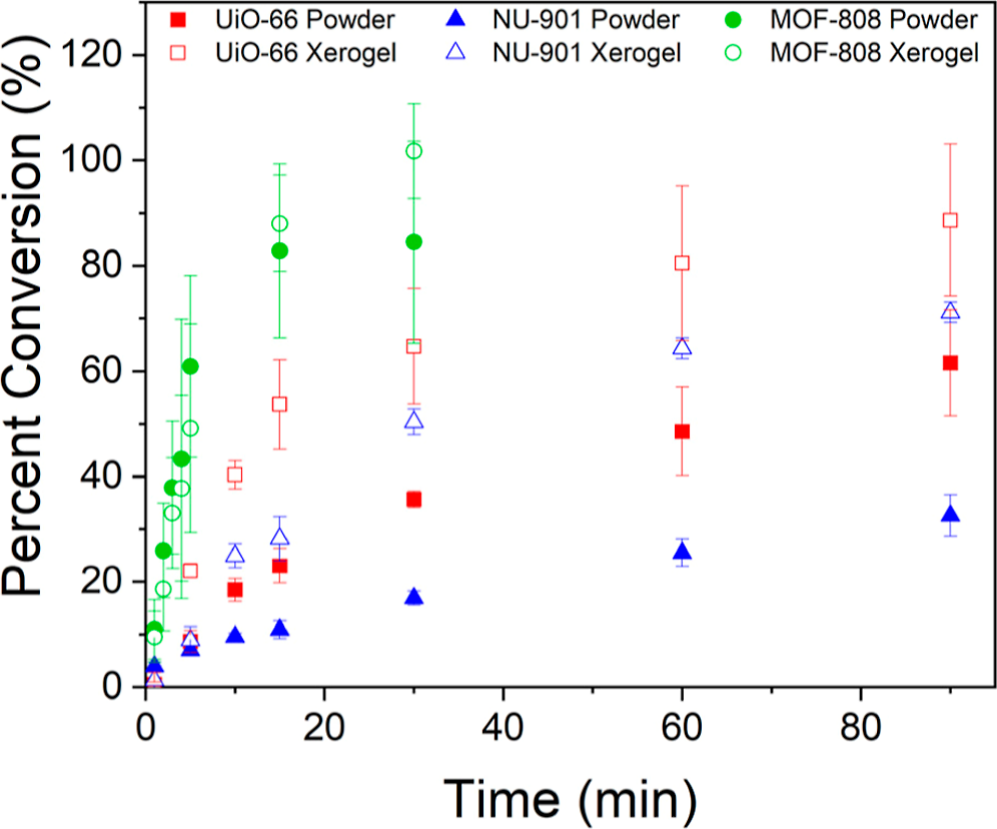
Percent conversion of DMNP based on total hydrolysis product (4-nitrophenol +4-nitrophenolate) over time for each MOF powder (filled) and xerogel (hollow). Error bars = 1 standard deviation.

The activity of MOF xerogels for CWA hydrolysis was tested by using DMNP as a nerve-agent simulant. Hydrolysis of DMNP was conducted by adding 5 mg of MOF powder or xerogel to a 0.45 M *N*-ethylmorpholine solution (buffered to pH 7 with acetic acid) containing 4 μL of DMNP. Aliquots of the solution were taken at set time intervals for analysis via electronic absorption spectroscopy. The hydrolysis of DMNP was monitored by the decrease in a peak at 279 nm, corresponding to DMNP and the growth of peaks at 404 and 313 nm, corresponding to hydrolysis products 4-nitrophenolate and 4-nitrophenol, respectively (Figures S10–S15). All of the xerogels were competent in DMNP hydrolysis, with the UiO-66 (10 ± 1 mmol/s·g) and NU-901 xerogels (5.5 ± 0.6 mmol/s·g) noticeably outperforming their powder counterparts (4.4 ± 0.6 and 2.2 ± 1.9 mmol/s·g, respectively) (Figures 6, S16–S18, and Table 2). The results support the hypothesis that the higher defect levels and external surface area of the xerogels relative to those of the powders should increase reactivity. Previous reports indicated that DMNP hydrolysis in MOFs with small pore apertures is largely surface limited; therefore, increasing the number of nodes at the surface is expected to significantly increase reactivity. Additionally, the presence of large mesopores in UiO-66 and NU-901 xerogels will significantly increase the access to catalytic sites.

**Table 2:** Initial Rates of DMNP Hydrolysis for Each MOF Powder and Xerogel Determined for the First 4 Time Points, and the Percent Conversion for Each MOF at the Final Measured Timepoint

MOF	rate (mmol/s·g)	percent conversion at final time point (%)
MOF-808 powder	33 ± 13	84 ± 19
MOF-808 xerogel	27 ± 11	102 ± 9
UiO-66 powder	4.4 ± 0.6	62 ± 10
UiO-66 xerogel	10 ± 1	89 ± 14
NU-901 powder	2.2 ± 1.9	32.5 ± 2
NU-901 xerogel	5.5 ± 0.6	71 ± 2

The MOF-808 xerogel and powder exhibit rates within error of each other (27 ± 11 and 33 ± 13 mmol/s·g, respectively). MOF-808 has long been known as one of the best MOFs for DMNP hydrolysis due to its low coordination number resulting in a greater number of active sites and its large pore size, allowing for easier diffusion of DMNP to active sites.^29^ However, one factor that is not often considered is the density of active sites per unit area in a given MOF structure. In native MOF-808, the size and geometry of pores directed by the 1,3,5-BTC linker results in the largest density of open metal sites/Å^3^ (2.12 × 10^–3^ open metal sites/Å^3^) out of the three MOFs studied here (1.80 × 10^–3^ for UiO-66 and 5.24 × 10^–4^ for NU-901), increasing the active sites available for hydrolysis (Figures S19–S21 and Table S2). While the MOF-808 xerogel might be expected to perform better than the microcrystalline powder, the surface area and ^1^H NMR analysis for the MOF-808 xerogel explain the disparity. Much of the additional surface area and added mesopores characteristic of the gels are inaccessible due to the overabundance of additional linkers. The excess linker is most likely bound to surface nodes, blocking the otherwise open sites in the native MOF-808 powder. Thus, no net enhancement of reactivity is observed between powder and xerogel MOF-808 like in the other two MOFs.

The powders and xerogels, except for NU-901 powder, display turnover numbers (TON) greater than 1 after only 15 min (Table S3), indicating catalytic behavior. The turnover frequency (TOF) or TON over time, is also calculated and shows similar behavior to the trends observed in rate calculations, with the UiO-66 and NU-901 xerogels outperforming the powders while MOF-808 powder and xerogel remaining within error. The percent conversion was calculated for each MOF (Table 2), with the MOF-808 xerogel achieving full conversion at 30 min. UiO-66 xerogel and MOF-808 powder also approach full conversion at the final time point of measurement.

In order to confirm that the xerogels retain their MOF structure and are not degraded under catalytic conditions, the MOFs were collected from the reaction solution by centrifugation, washed with water and acetone, and analyzed by PXRD (Figures S22–S24). In all cases, the peak positions and relative intensity are maintained postcatalysis. Particle morphology and crystallite size (as determined by the Scherrer equation) of the gels before and after reactivity were also maintained (Figure S25). The SEM of NU-901 xerogel shows much more definition in the particles, as well as larger particles on average (ca. 95 nm compared to 45 nm precatalysis) (Figure S25, right). NU-901 xerogel displayed the same size increase when it was simply subjected to *N*-ethylmorpholine solution in the absence of DMNP (Figure S26). The change in particle size and definition would suggest growth of the particles in the presence of water and/or base, though these particles are still roughly 2 orders of magnitude smaller than the powder particles. SEM of the UiO-66 and MOF-808 xerogels postcatalysis are still composed of largely undefined particles, but sharper corners or edges can be seen sporadically throughout the SEM, which could suggest a possible Oswald ripening while in solution. Ideally, further experiments on the postreaction solution would be performed to monitor for MOF degradation products such as linker or zirconium ions. However, due to the handling requirements of DMNP, these experiments cannot be readily performed. That said, the available results indicate the MOF xerogels are generally stable to the catalytic conditions used here.

**Figure 7:**
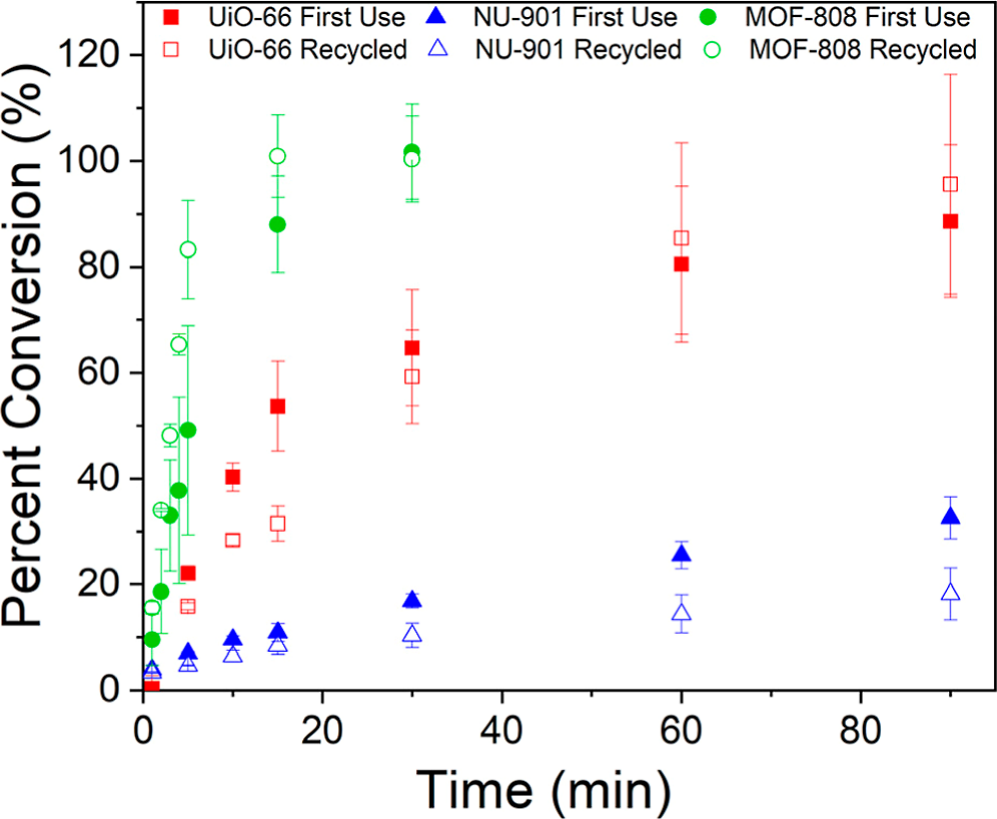
Percent conversion of DMNP based on total hydrolysis product (4-nitrophenol +4-nitrophenolate) over time for each MOF xerogel. First use = filled and recycled = hollow. Error bars = 1 standard deviation.

To demonstrate the recyclability of the xerogel catalysts, DMNP hydrolysis experiments were carried out with the used xerogel powders (Figures 7 and S27–S29). UiO-66 xerogel remains largely consistent upon recycling at longer time periods, with only a minor decrease in initial rate. NU-901 xerogel showed a slight loss in reactivity upon reuse, with both lower total rates and TON (Tables 3 and S4). This loss of reactivity is likely due to a combination of the larger particles and capping of zirconium nodes with dimethyl hydrogen phosphate (DHP), the phosphorus-containing product of the first hydrolysis reaction. The presence of this byproduct was confirmed by ^31^P NMR of all the xerogels post catalysis (Figures S30–S32). The coordination of DHP to zirconium nodes is reported to be highly stable due to DHP forming a bridge between two zirconium atoms in the node,^50^ effectively blocking these sites from participating in hydrolysis. This hypothesis is further supported by a noticeable discoloration of all MOF xerogels postreaction.

**Table 3:** Initial Rates of DMNP Hydrolysis for Each Recycled MOF Xerogel Determined for the First 4 Time Points, and the Percent Conversion for Each MOF at the Final Measured Timepoint

MOF	rate (mmol/s·g)	percent conversion at final time point (%)
MOF-808 xerogel	43.8 ± 0.2	100 ± 8
UiO-66 xerogel	6.4 ± 0.3	96 ± 21
NU-901 xerogel	3.6 ± 0.1	54 ± 1

Interestingly, the MOF-808 xerogel had a higher overall performance upon reuse. The rate of the reused material (43.8 ± 0.2 mmol/s·g) is over 1.5 times that seen in the first use of the xerogel (27 ± 11 mmol/s·g) and reaches 100% conversion in nearly half the time of MOF-808 powder. The reason for this substantial increase in activity is likely additional available active sites in the MOF-808 xerogel upon recycling, which is supported by ^1^H NMR of the recycled material that shows a decrease in the excess linker present compared to the as-synthesized sample (Figure S33). Attempts to purposely remove excess linkers by treating MOF-808 xerogel with HCl were also successful in decreasing the amount of linker present (Figure S34), but no noticeable change in activity was observed (Figure S35). A second HCl treatment resulted in lower overall conversion. Further work to replicate the recycling procedure in as-synthesized MOF-808 xerogel, such as MeOH reflux, high temperature and pressure washing, and soaking at pH 10 results in loss of linker but no significant improvement in reactivity (Figure S36). Further studies are needed to understand structural changes that occur during initial catalytic experiments that result in an improved material.

## 3 Conclusions

We synthesized highly porous xerogels based on three commonly reported Zr-based MOFs: UiO-66, MOF-808, and NU-901. The xerogels retained the crystal structure of the MOFs in powder form, while N_2_ isotherms revealed mesoporosity not present in MOF powders. The MOF xerogels appeared to be composed of closely packed, highly defected MOF nanocrystals (<50 nm) arranged in aggregate networks with interparticle pores 100–400 Å in size, and these MOF xerogels were highly active for hydrolysis of DMNP, with UiO-66 and NU-901 demonstrating significant enhancement over their native powders. MOF-808 remains more active than both UiO-66 and NU-901 in xerogel and powder form due to the low coordination of the framework and the high density of active sites per unit area. Recycled MOF-808 xerogel displays the shortest time to 100% conversion of DMNP of any of the studied MOFs and is among the most rapid catalysts for DMNP hydrolysis studied to date. Based on the SEM and surface area calculations, we believe the increase in reactivity is largely driven by the additional external surface area. The formation of the MOF xerogel provides significantly more access to reactive sites compared to the powders, owing to its smaller particle size and the introduction of mesopores.

## Supporting Information Available

Additional synthetic details and characterization of materials including TGA, BET data tables, full UV–vis spectra of DMNP hydrolysis reactions, TON/TOF tables, and PXRD post catalysis (PDF)
