# Supplementary material for: Macromorphological Control of Zr-Based Metal–Organic Frameworks for Hydrolysis of a Nerve Agent Simulant
Source: ACS Appl Mater Interfaces. 2024 Sep 18;16(39):52703–11. doi: 10.1021/acsami.4c11928 (PMC11450694; doi:10.1021/acsami.4c11928)
Supplement: Supplementary file 1 — am4c11928_si_001.pdf [file am4c11928_si_001.pdf]

Supporting Information

Macromorphological Control of Zr-based Metal-Organic Frameworks for Hydrolysis of a Nerve Agent Simulant

Bradley Gibbons ‡, Eric M. Johnson ‡, Mohammad Khurram Javed, Xiaozhou Yang, and Amanda J. Morris*

Department of Chemistry, Virginia Tech, Blacksburg, VA, 24061, USA

* Corresponding Author:

Amanda J. Morris, Email: ajmorris@vt.edu

‡ These authors have contributed equally.

MATERIALS CHARACTERIZATION:

Powder X-ray Diffraction. PXRD of each sample was collected on a Rigaku Miniflex benchtop diffractometer (Cu Kα radiation $\lambda = 1.5418$ Å). The samples were analyzed at 0.1° resolution and a 15°/min continuous scanning mode from 3-50° 2θ.

Scanning Electron Microscopy. To measure particle size, SEM images were collected using a Leo/Zeiss 1550 field-emission scanning electron microscope. Samples for SEM were prepared on fluorine-doped tin oxide slides attached to SEM sample stages with copper tape and conductive adhesive. The MOF powder was suspended in ethanol and drop cast onto the slide.

Thermogravimetric Analysis. Defect characterization was collected with a TA instruments TGA-5500 TGA. For all samples 10-15 mg of MOF was loaded onto a Pt pan and ran under an air flow from 30-650 °C at a ramp rate of 10 °C/minute. Defect level was calculated following literature procedures.[1, 2]

^{1}H Nuclear Magnetic Resonance. Basic D_2O was prepared by dissolving 5 mg of NaOH into 1 mL of D_2O and sonicating briefly. To that solution, 20 mg of UiO-66 was mixed and allowed to soak overnight. The following day, the sample was filtered and analyzed by ^{1}H NMR.

EXPERIMENTAL SECTION:

$ZrCl_4$, $ZrOCl_2$•$8H_2O$, terephthalic acid, 1,3,5-benzenetricarboxylic acid, dimethylformamide (DMF), glacial acetic acid (AA), formic acid (FA), HCl, and benzoic acid were purchased from commercial suppliers and used as received. Trifluoroacetic acid (TFA) was purchased from commercial suppliers and dried over Drierite dessicant before use. 1,3,6,8-Tetrakis(p-benzoic acid)pyrene was prepared following literature procedures.[3]

Synthesis of UiO-66 Powder. UiO-66 powder was synthesized following reported procedures[2] by adding 86 mg of $ZrCl_4$ (0.37 mmol) and 61 mg of terephthalic acid (0.37 mmol) to 10 mL of DMF and 2.5 mL of glacial acetic acid in a 6-dram vial. The vials were capped and placed in an oven at 120 °C for 72 h. The white powder was centrifuged and washed with DMF (3× over 24 h) and acetone (3× over 24 h) to yield UiO-66 powder as a white powder.

Synthesis of UiO-66 Xerogel. UiO-66 xerogel was synthesized following reported procedures[4] by adding 3.22 g $ZrOCl_2$•$8H_2O$ (10 mmol), 2.41 g terephthalic acid (14.5 mmol), and 60 mL DMF to a 100 mL pyrex screw-cap bottle. Then 1.5 mL 37 wt% HCl and 2 mL glacial acetic acid were added, and the bottle was placed in an oven at 100 °C for 2 h. The resultant gel was washed with DMF at 120 °C overnight two times and ethanol at 60 °C overnight 3 times. The gel was then transferred to a 6-dram vial and dried in an oven at 200 °C for 2 h to form UiO-66 xerogel as a white powder.

Synthesis of MOF-808 Powder. MOF-808 powder was synthesized following reported procedures[5] by adding 9.7 g $ZrOCl_2$•$8H_2O$ (30 mmol), 2.1 g 1,3,5-benzenetricarboxylic acid (10

mmol), 450 mL DMF, and 450 mL formic acid in a 1 L pyrex screw-cap bottle. The mixture was sonicated well for 15 min and put in an oven at 130 °C for 48 h. The white powder was centrifuged and washed with DMF (3× over 24 h) and acetone (3× over 24 h) to yield MOF-808 powder as a white powder.

Synthesis of MOF-808 Xerogel. MOF-808 xerogel was synthesized following reported procedures[4] by adding 1.07 g $ZrOCl_2•8H_2O$ (3.3 mmol), 1.02 g 1,3,5-benzenetricarboxylic acid (4.8 mmol), 10 mL DMF, 10 mL formic acid, and 0.3 mL distilled water to a 100 mL pyrex screw-cap bottle. The bottle was placed in an oven at 100 °C for 2 h. The resultant gel was washed with DMF at 120 °C overnight two times and ethanol at 60 °C overnight 3 times. The gel was then transferred to a 6-dram vial and dried in an oven at 200 °C for 2 h to form MOF-808 xerogel as white powder.

Synthesis of NU-901 Powder: NU-901 powder was synthesized following reported procedures[6] by adding 97 mg $Zr(acac)_4$ (0.2 mmol) and 3.02 g 4-aminobenzoic acid (22 mmol) to 8 mL of DMF in a 6 dram vial. The solution was incubated in an oven at 80 ^{0}C for 1 h. 40 mg of H4TBAPy (0.06 mmol) linker was then added and sonicated until completely dissolved. The resultant yellow solution was then placed in a pre-heated oven at 100 ^{0}C for 18 h. The yellow powder was centrifuged and washed with DMF (3× over 24 h) and methanol (3× over 24 h) to yield NU-901 powder (yield: ~30 mg).

DMNP Hydrolysis. To a 2-dram vial, 1 mL of a 0.45 M N-ethylmorpholine solution (aqueous, pH adjusted to 7 with glacial acetic acid) was added along with 4 µL of DMNP. The mixture was stirred at room temperature for ~5 minutes before adding 5 mg of MOF powder. To monitor the reaction progress, 20 µL aliquots of the reaction mixture were taken at 1, 5, 10, 15, 30, 60, and 90 minutes for UiO-66 and NU-1000 and 1, 2, 3, 4, 5, 15, and 30 minutes for MOF-808. The aliquots were diluted into 10 mL of 0.45 M N-ethylmorpholine solution adjusted to pH=7. Reaction progress was monitored by the absorption of 4-nitrophenolate (λ_{max}=404 nm) and 4-nitrophenol (λ_{max}=313 nm) on a Cary-5000 UV-VIS spectrometer.

At pH 7, both 4-nitrophenol and 4-nitrophenolate appear as degradation products with somewhat overlapping peaks. To properly account for both products, peaks were fit using Origin (Figure S1). The max absorbance of the fit peaks was then converted to concentrations using a calibration plot of 4-nitrophenol (HNP) and 4-nitrophenolate (NP-) (Figures S2-S4). The amounts of 4-nitrophenol and 4-nitrophenolate were summed to get the total amount of degraded DMNP.

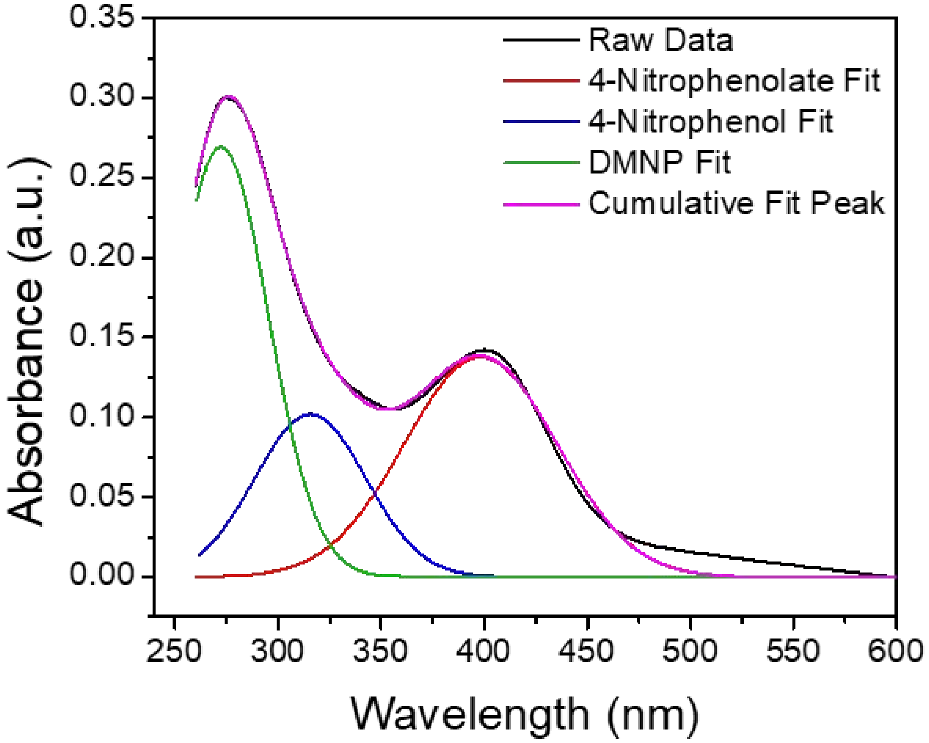

Figure S1. Example of a fit UV-Vis spectrum at a single time point where peaks are fit to DMNP (green), 4-nitrophenol (blue), and 4-nitrophenolate (red). The summed fit peaks (magenta) match the raw data (black) in the region of interest.

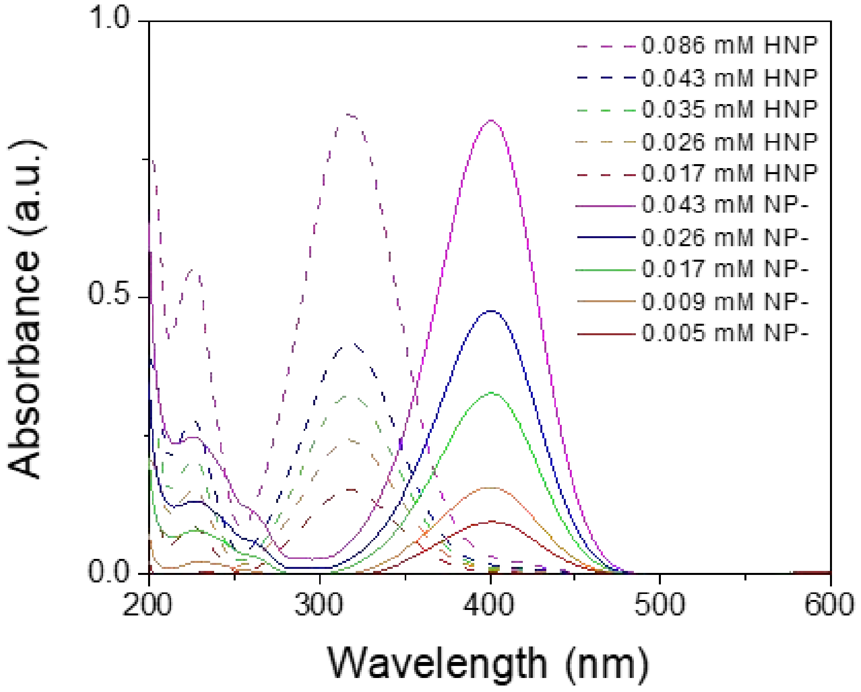

Figure S2. UV-Vis of 4-nitrophenol (HNP) and 4-nitrophenolate (NP-) at varied concentrations used to construct calibration plots for molar absorptivity calculations.

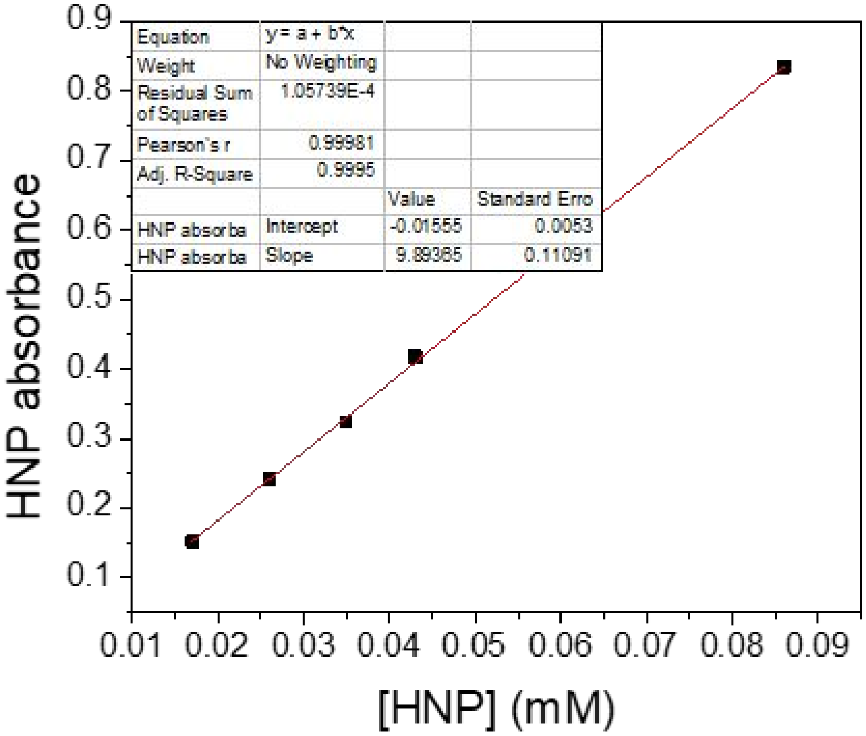

Figure S3. Calibration plot for 4-nitrophenol, where the slope of 9.89 $mM^{-1}cm^{-1}$ is the molar absorptivity.

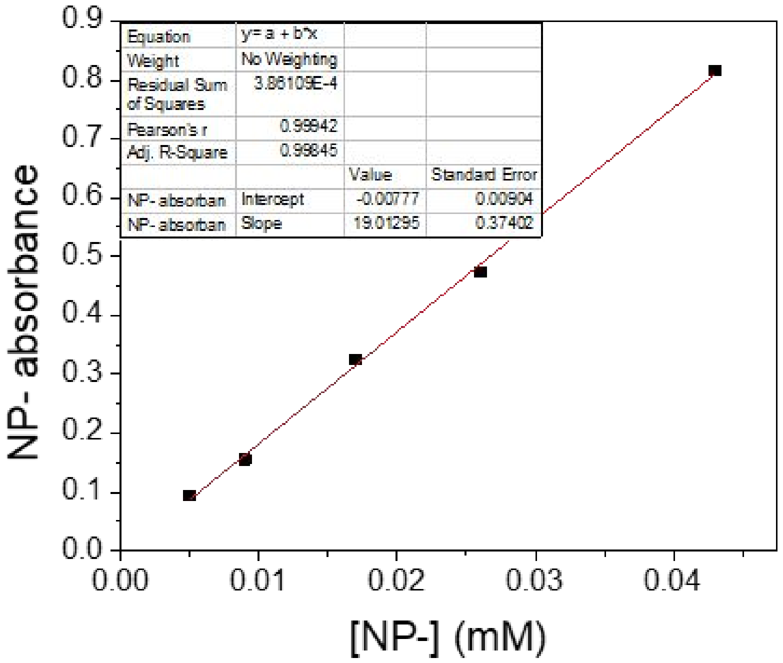

Figure S4. Calibration plot for 4-nitrophenolate, where the slope of 19.0 $mM^{-1}cm^{-1}$ is the molar absorptivity.

ADDITIONAL INFORMATION:

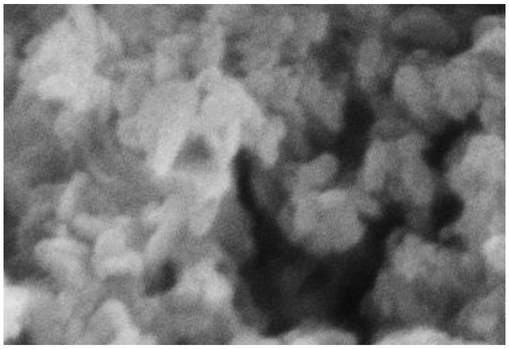

Figure S5. Zoomed in SEM of NU-901 xerogel.

Table S1. BET surface area, relative number of mesopores, and percent external surface area for each MOF powder and xerogel.

Sample	BET Surface Area (m^2/g)	V_{meso}/V_{micro}	% External Surface Area
UiO-66 powder	1402	0.13	14.57
UiO-66 xerogel	1513	2.87	22.93
MOF-808 powder	1652	0.30	18.25
MOF-808 xerogel	450	3.12	47.23
NU-901 powder	1777	0.71	29.13
NU-901 xerogel	1844	6.31	63.77

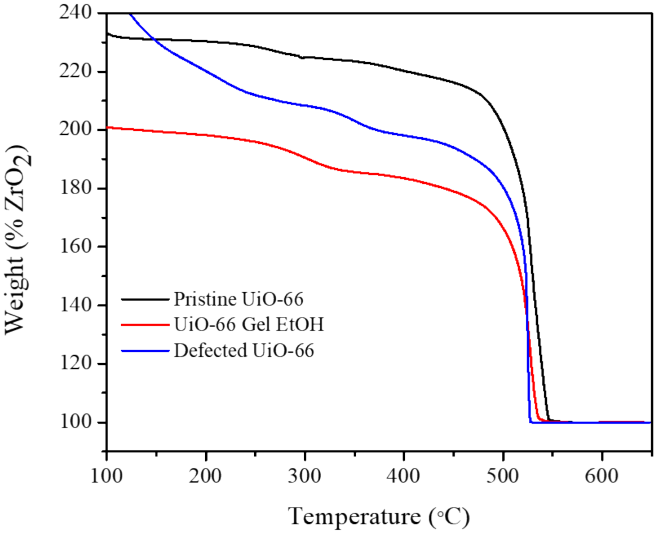

Figure S6. TGA of pristine UiO-66 (black), UiO-66 xerogel (red), and UiO-66 powder (blue) run under air.

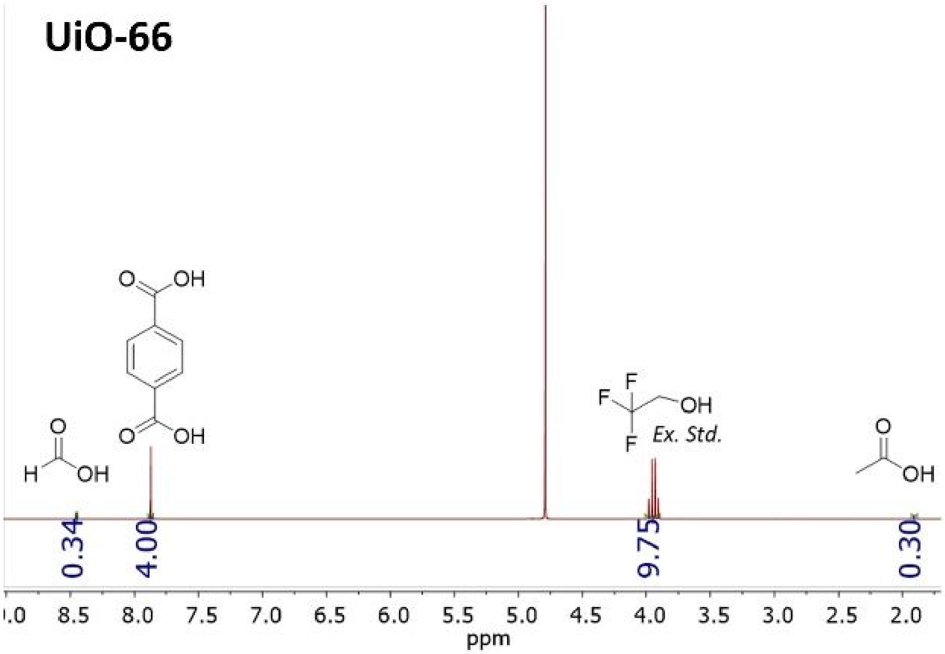

Figure S7. ^{1}H NMR (400 MHz) of digested UiO-66 xerogel in basic D_2O.

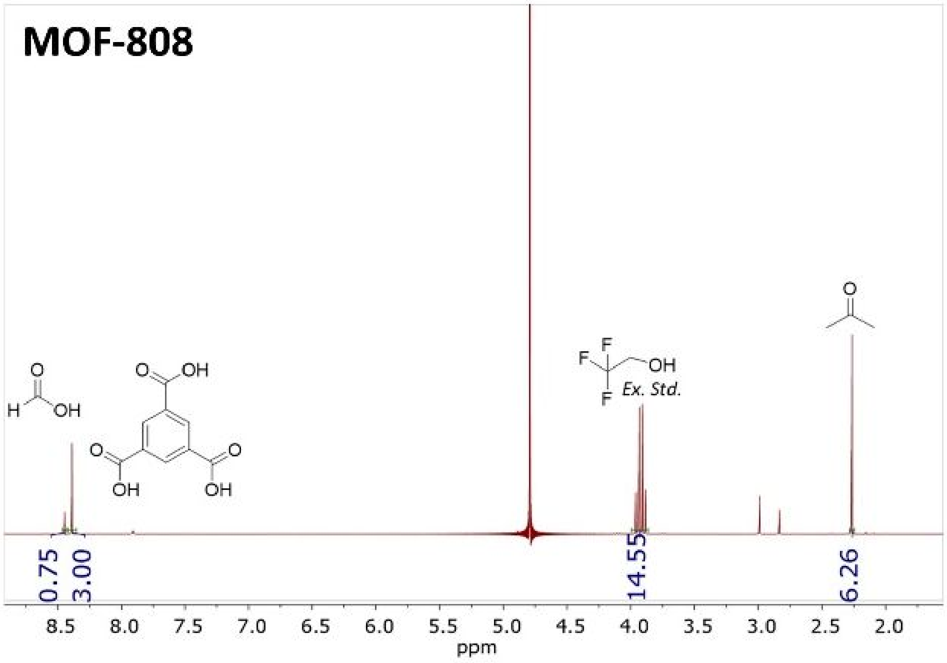

Figure S8. ^{1}H NMR (400 MHz) of digested MOF-808 xerogel in basic D_2O.

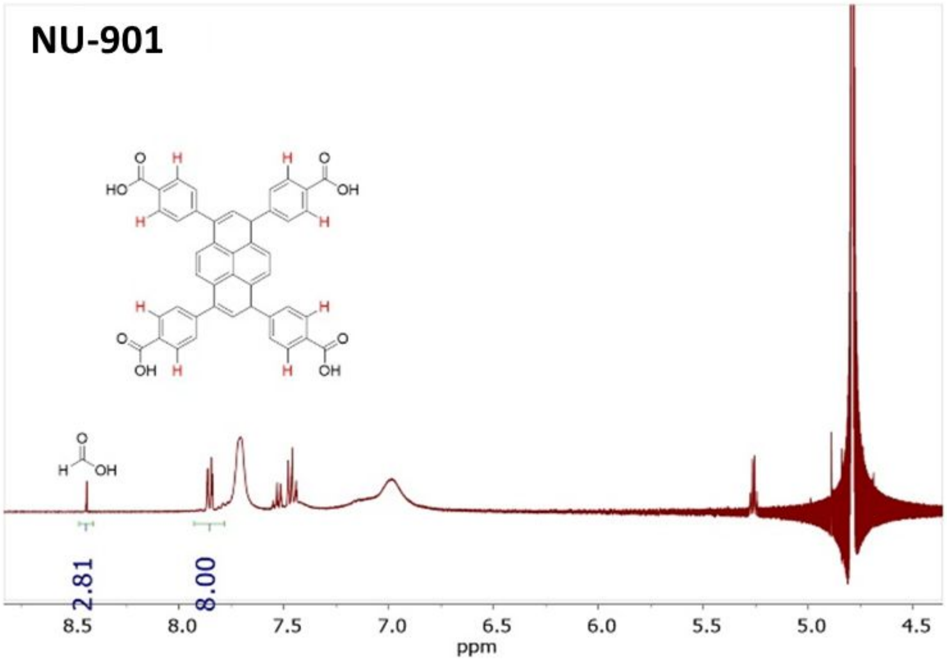

Figure S9. ^{1}H NMR (400 MHz) of digested NU-901 xerogel in basic D_2O.

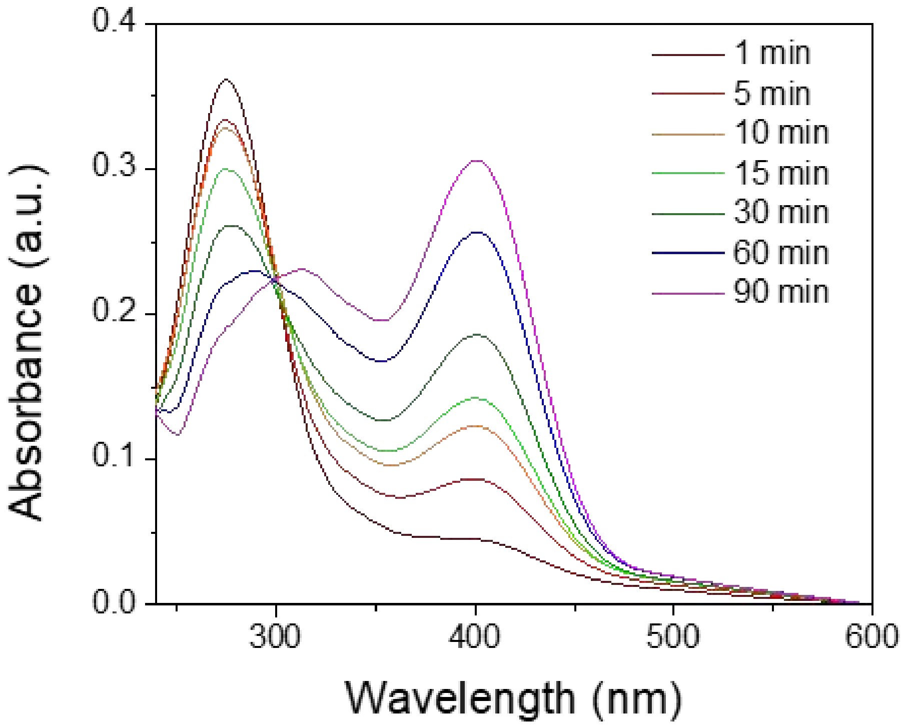

Figure S10. UV-Vis of DMNP hydrolysis experiment performed with UiO-66 powder.

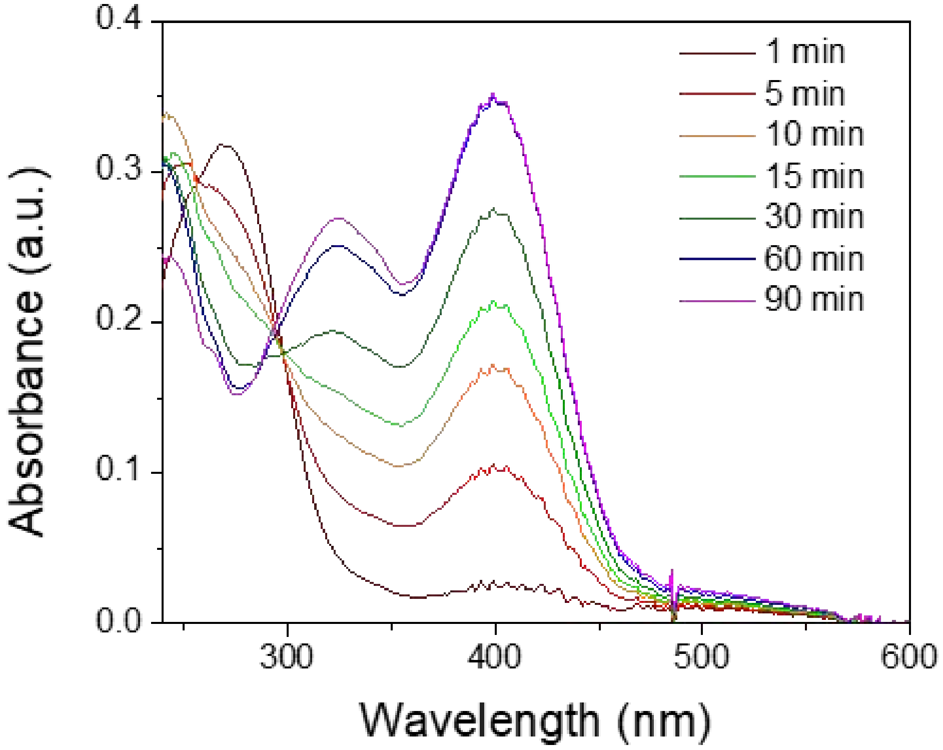

Figure S11. UV-Vis of DMNP hydrolysis experiment performed with UiO-66 xerogel.

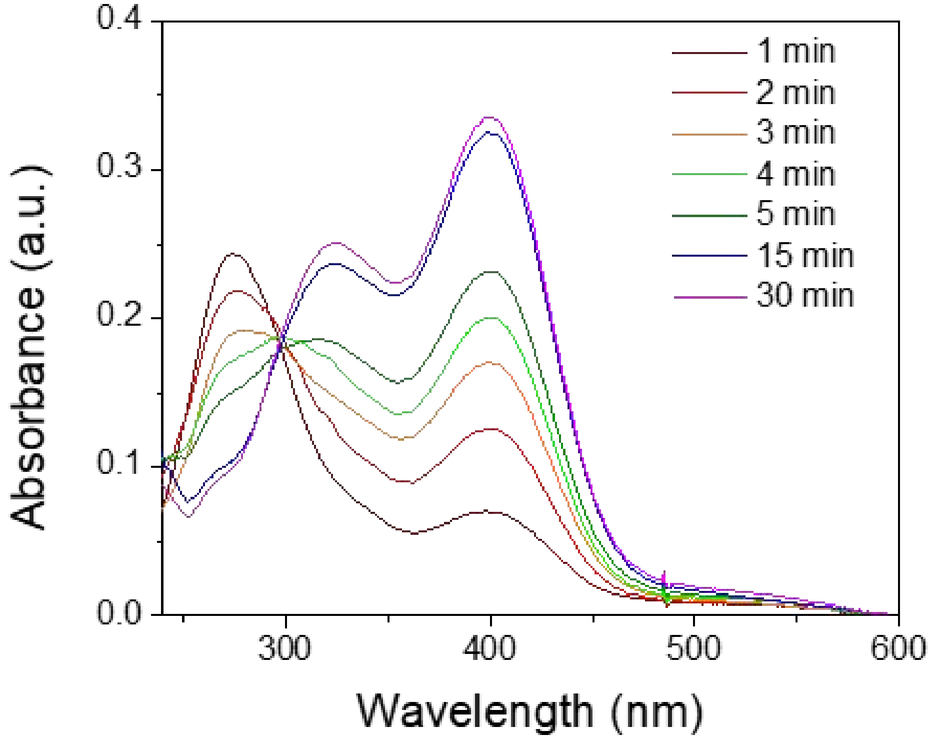

Figure S12. UV-Vis of DMNP hydrolysis experiment performed with MOF-808 powder.

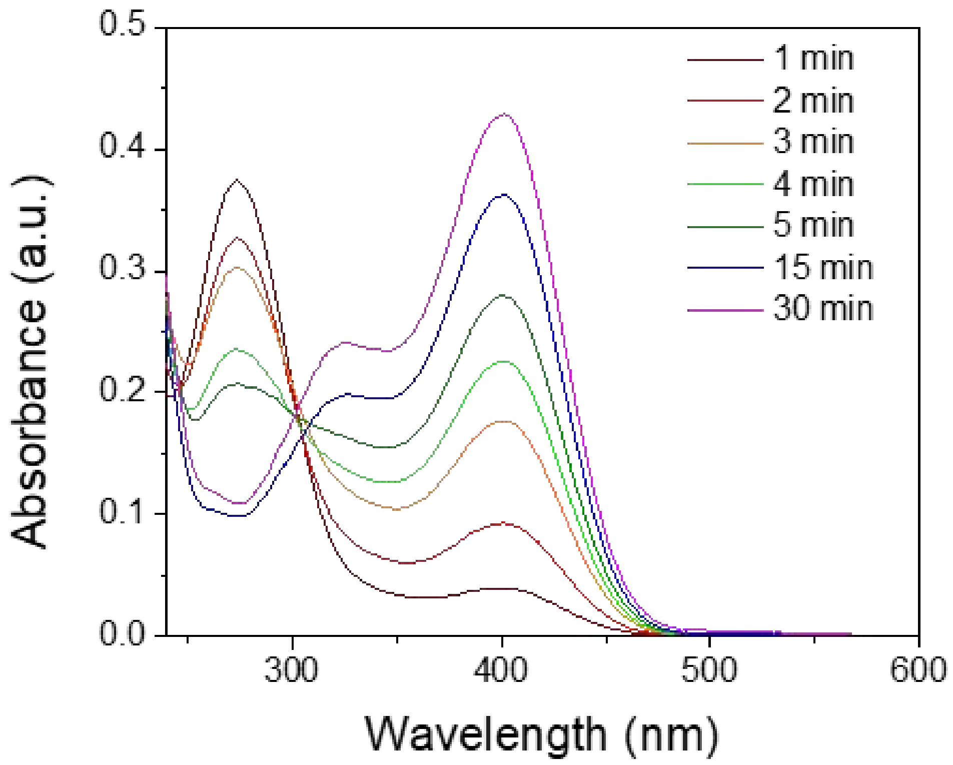

Figure S13. UV-Vis of DMNP hydrolysis experiment performed with MOF-808 xerogel.

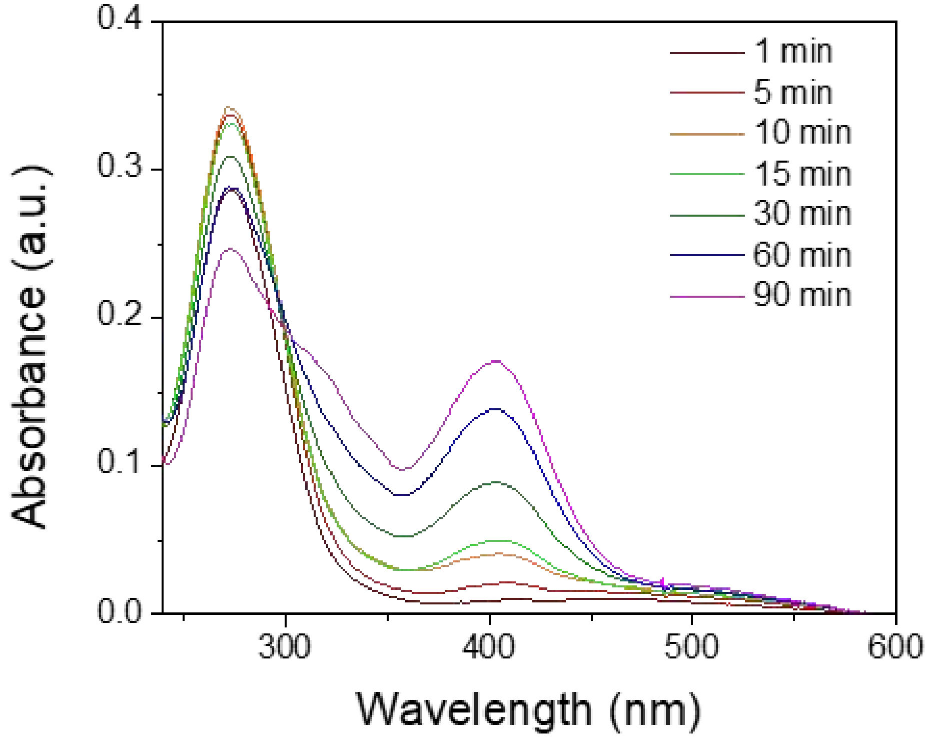

Figure S14. UV-Vis of DMNP hydrolysis experiment performed with NU-1000 powder.

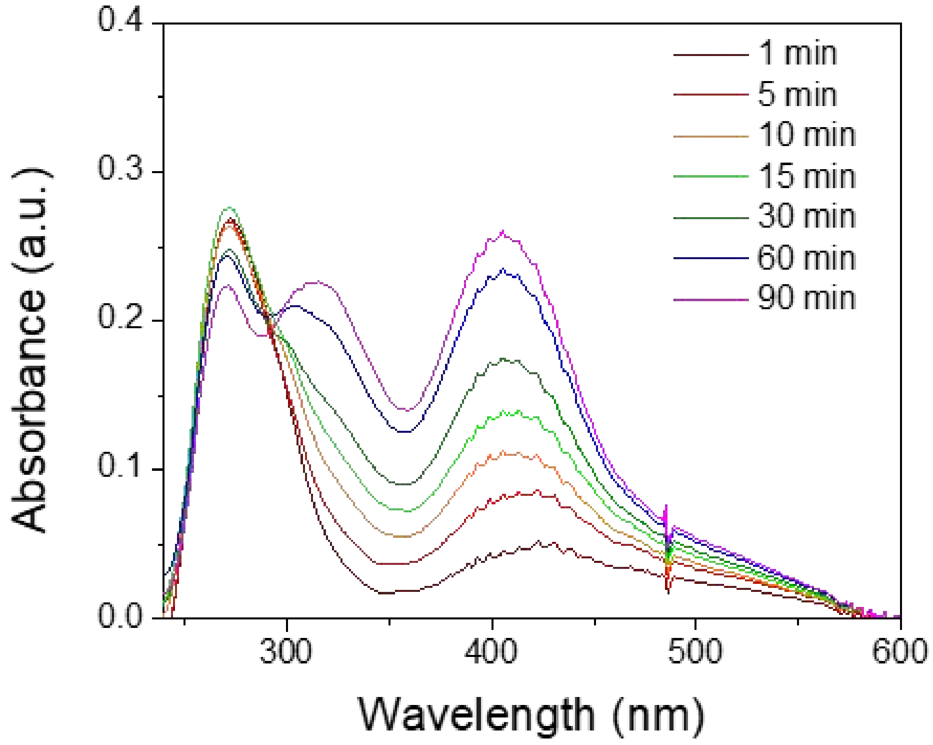

Figure S15. UV-Vis of DMNP hydrolysis experiment performed with NU-901 xerogel.

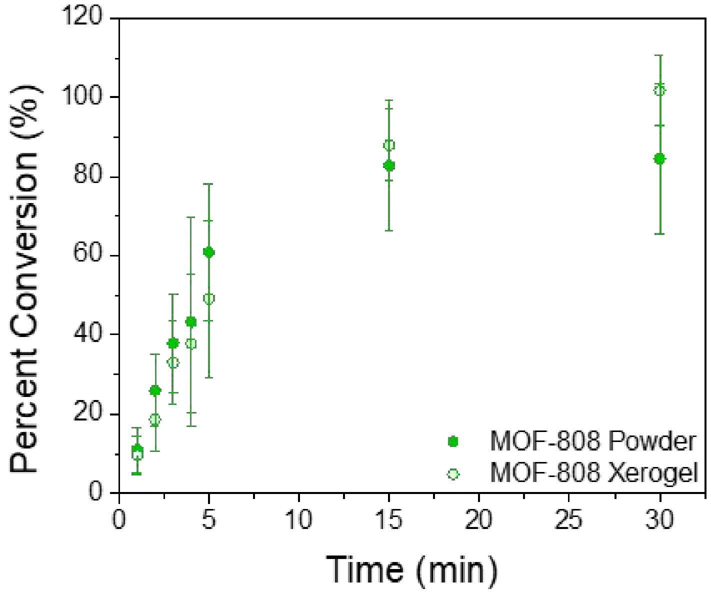

Figure S16. Percent conversion of DMNP based on total hydrolysis product (4-nitrophenol + 4-nitrophenolate) over time for MOF-808 powder (filled) and xerogel (hollow). Error bars = 1 standard deviation.

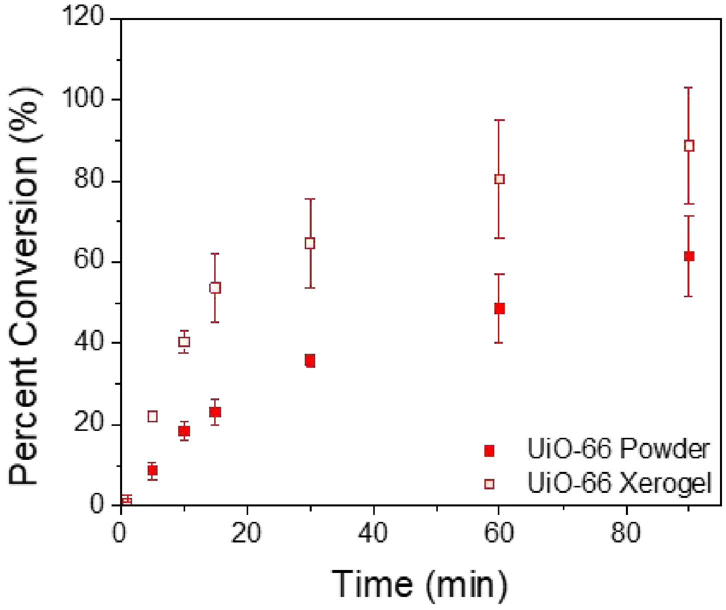

Figure S17. Percent conversion of DMNP based on total hydrolysis product (4-nitrophenol + 4-nitrophenolate) over time for UiO-66 powder (filled) and xerogel (hollow). Error bars = 1 standard deviation.

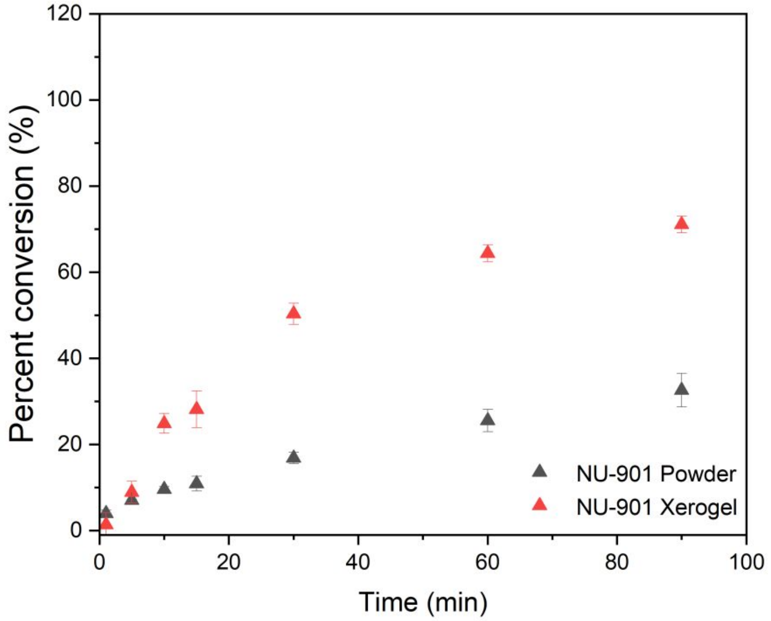

Figure S18. Percent conversion of DMNP based on total hydrolysis product (4-nitrophenol + 4-nitrophenolate) over time for NU-901 powder (filled) and xerogel (hollow). Error bars = 1 standard deviation.

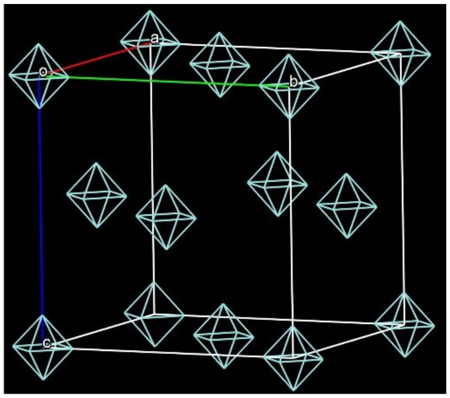

UiO-66

$$\#Nodes = \left(6\ Face\ Nodes \times \frac{1}{2}\right) + \left(8\ Corner\ Nodes \times \frac{1}{8}\right)$$

4 Nodes per Unit Cell
Unit Cell Volume = 8870.26 Å^3

Figure S19. Crystal structure of UiO-66 with only the Zr_6 nodes shown along with the calculation of number of nodes per unit cell.

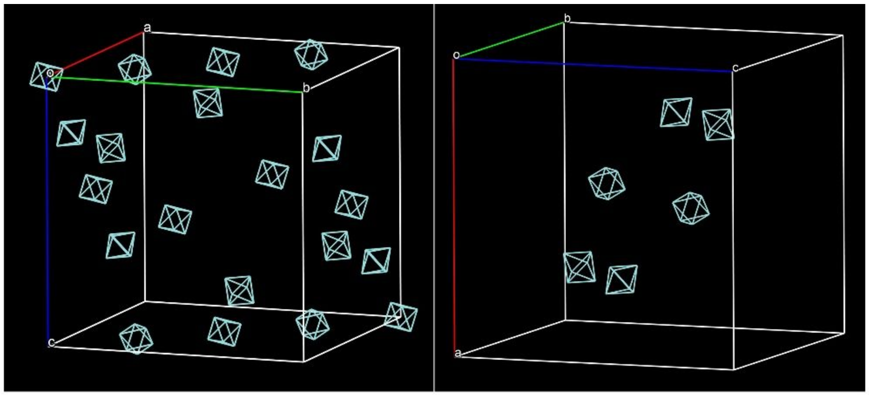

MOF-808

Exterior Nodes (Left), Interior Nodes (Right)

$$\#Nodes = \left(18\ Face\ Nodes \times \frac{1}{2}\right) + \left(2\ Corner\ Nodes \times \frac{1}{8}\right) + 6\ Interior\ Nodes$$

15.25 Nodes per Unit Cell
Unit Cell Volume = 43156.4 Å^3

Figure S20. Crystal structure of MOF-808 with only the Zr_6 nodes shown along with the calculation of number of nodes per unit cell.

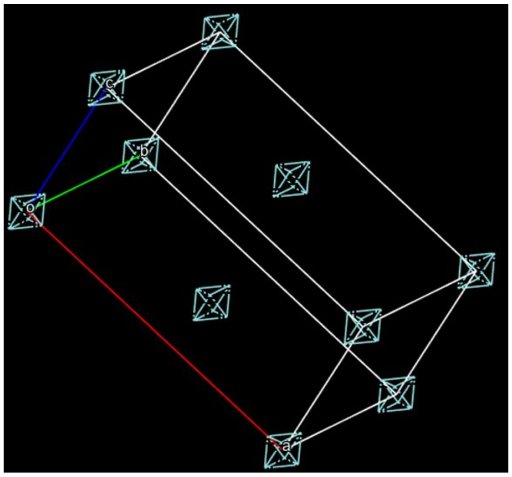

NU-901

$$\#Nodes = \left(2\, Face\ Nodes\ \times \frac{1}{2} + 8\, Corner\ Nodes\ \times \frac{1}{8}\right)$$

2 Nodes per Unit Cell
Unit cell volume = 7177.91 Å

Figure S21. Crystal structure of NU-901 with only the Zr_6 nodes shown along with the calculation of number of nodes per unit cell.

Table S2. Table of Nodes/Unit Cell, Vacant Sites/Node, and Active Sites/Å^3.

	Nodes/Unit Cell	Vacant Sites/Node*	Active Sites/Å^3
UiO-66	4	4	1.80×10^{-3}
MOF-808	15.25	6	2.12×10^{-3}
NU-901	2	4	1.11×10^{-3}

*Vacant sites/node calculated assuming a full coordination of 12/node and based on TGA data for UiO-66. MOF-808 and NU-901 contain unsaturated nodes and their normal coordination number is used here.

Table S3. Turnover number (TON) and turnover frequency (TOF) for all MOF xerogels and powders.

	TON (15 min)	TOF (min^{-1}) (15 min)	TON (final time point)	TOF (min^{-1}) (final time point)
MOF-808 Powder	5.1	0.337	5.2	0.172
MOF-808 Xerogel	5.4	0.358	6.2	0.207
UiO-66 Powder	1.7	0.113	4.5	0.050
UiO-66 Xerogel	3.9	0.262	6.5	0.072
NU-901 Powder	0.8	0.059	3.5	0.039
NU-901 Xerogel	2.7	0.183	6.9	0.077

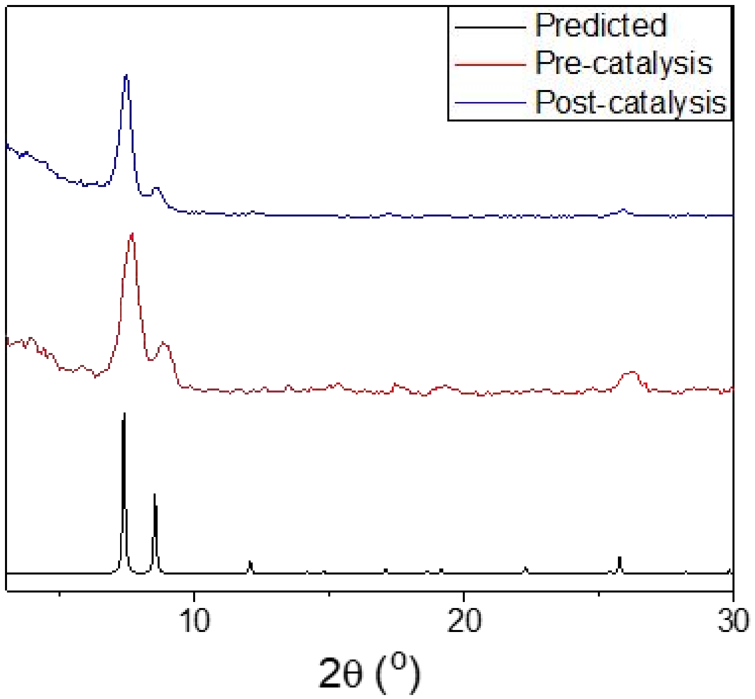

Figure S22. PXRD of UiO-66 xerogel before (red) and after (blue) use in DMNP hydrolysis reaction.

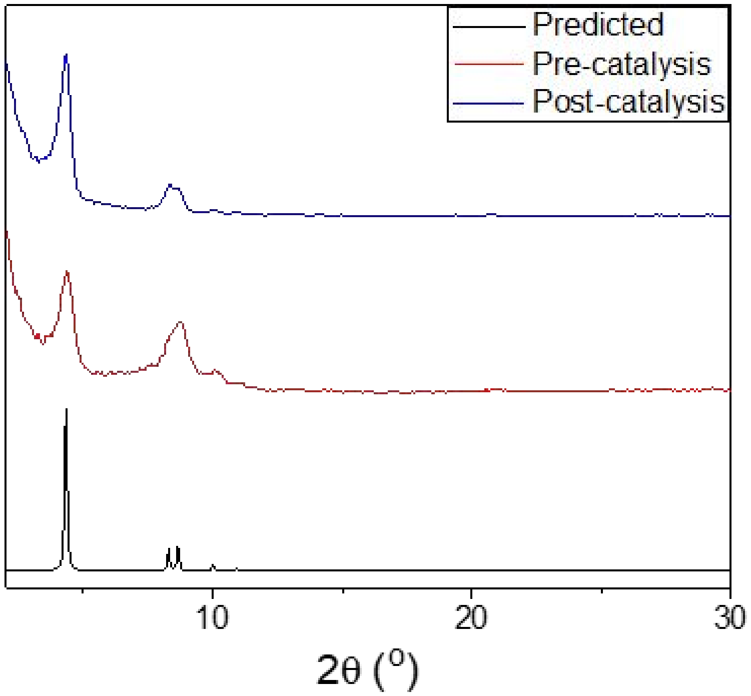

Figure S23. PXRD of MOF-808 xerogel before (red) and after (blue) use in DMNP hydrolysis reaction.

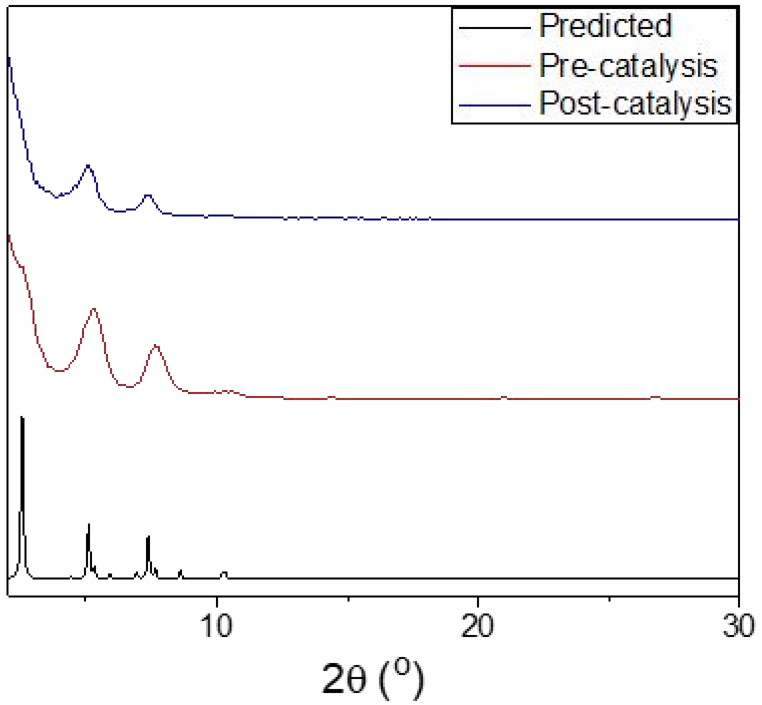

Figure S24. PXRD of NU-901 xerogel before (red) and after (blue) use in DMNP hydrolysis reaction.

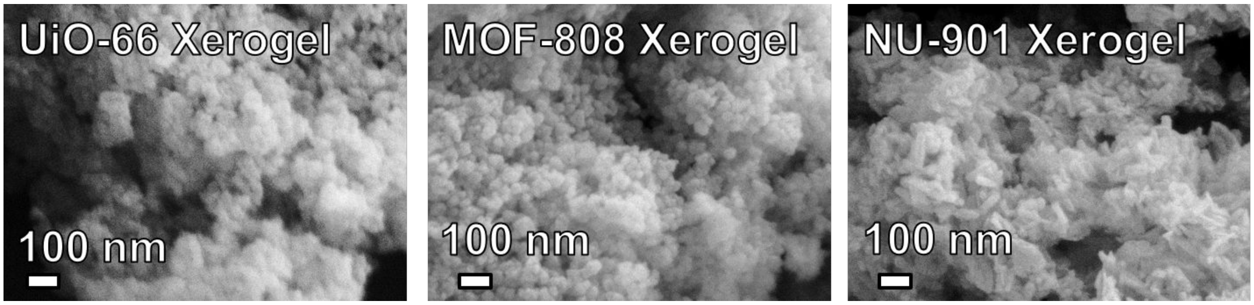

Figure S25. SEM of recycled UiO-66 (left), MOF-808 (middle), and NU-901 (right) xerogels.

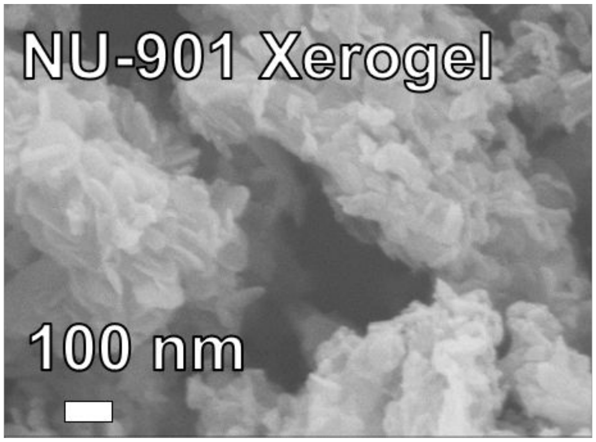

Figure S26. SEM of NU-1000 xerogel after being subjected to N-ethylmorpholine solution.

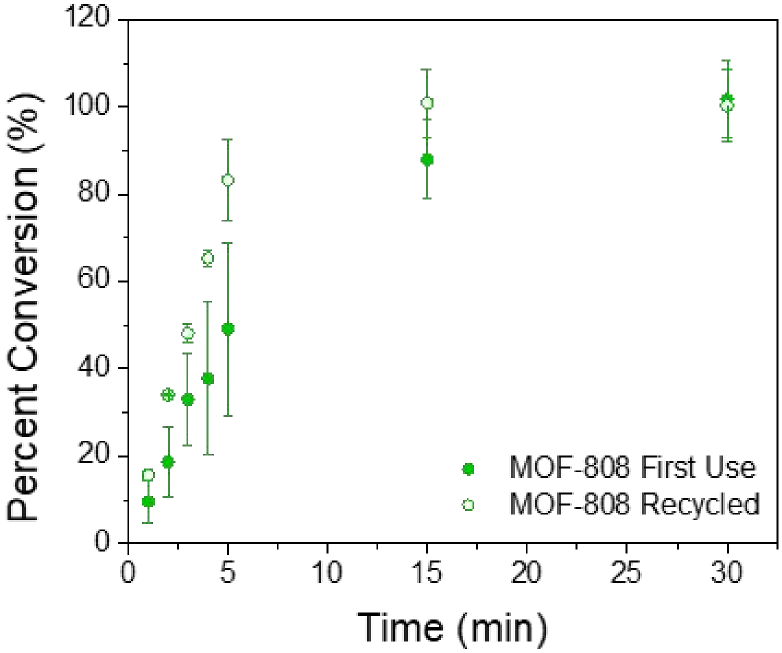

Figure S27. Percent conversion of DMNP based on total hydrolysis product (4-nitrophenol + 4-nitrophenolate) over time for MOF-808 xerogel. First use = filled and recycled = hollow. Error bars = 1 standard deviation.

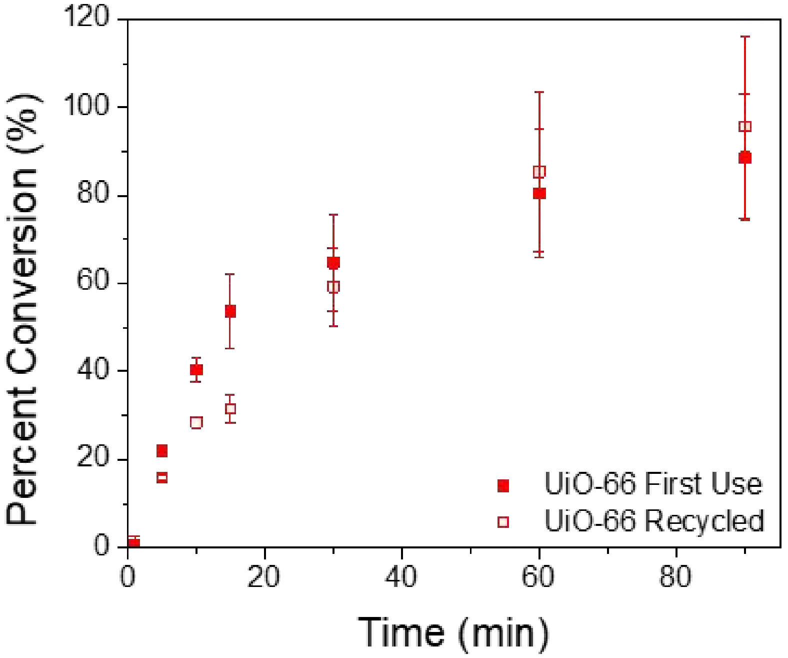

Figure S28. Percent conversion of DMNP based on total hydrolysis product (4-nitrophenol + 4-nitrophenolate) over time for UiO-66 xerogel. First use = filled and recycled = hollow. Error bars = 1 standard deviation.

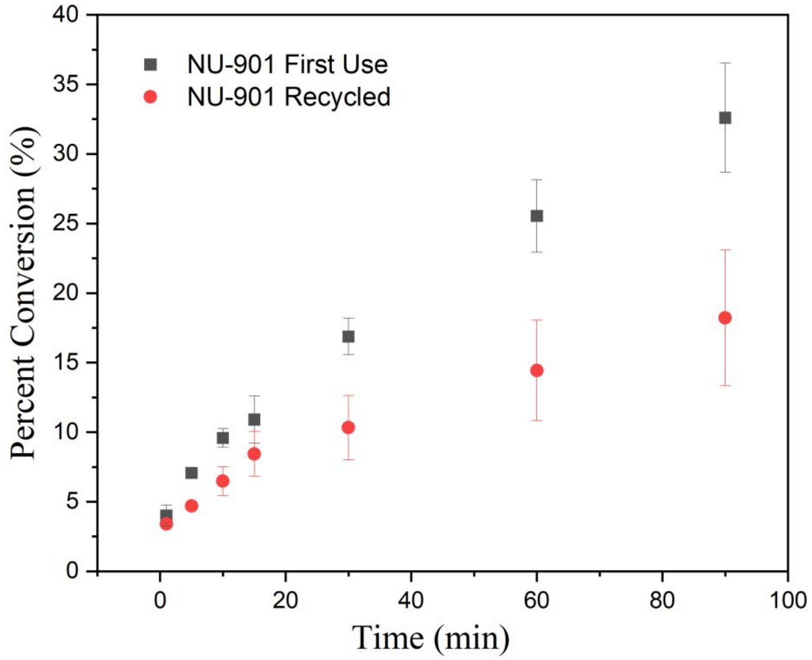

Figure S29. Percent conversion of DMNP based on total hydrolysis product (4-nitrophenol + 4-nitrophenolate) over time for NU-901 xerogel. First use = filled and recycled = hollow. Error bars = 1 standard deviation.

Table S4. Turnover number (TON) and turnover frequency (TOF) for all recycled MOF xerogels.

	TON (15 min)	TOF (min^{-1}) (15 min)	TON (final time point)	TOF (min^{-1}) (final time point)
MOF-808 Xerogel	6.2	0.411	6.1	0.204
UiO-66 Xerogel	2.3	0.154	7.0	0.078
NU-901 Xerogel	1.8	0.122	5.2	0.058

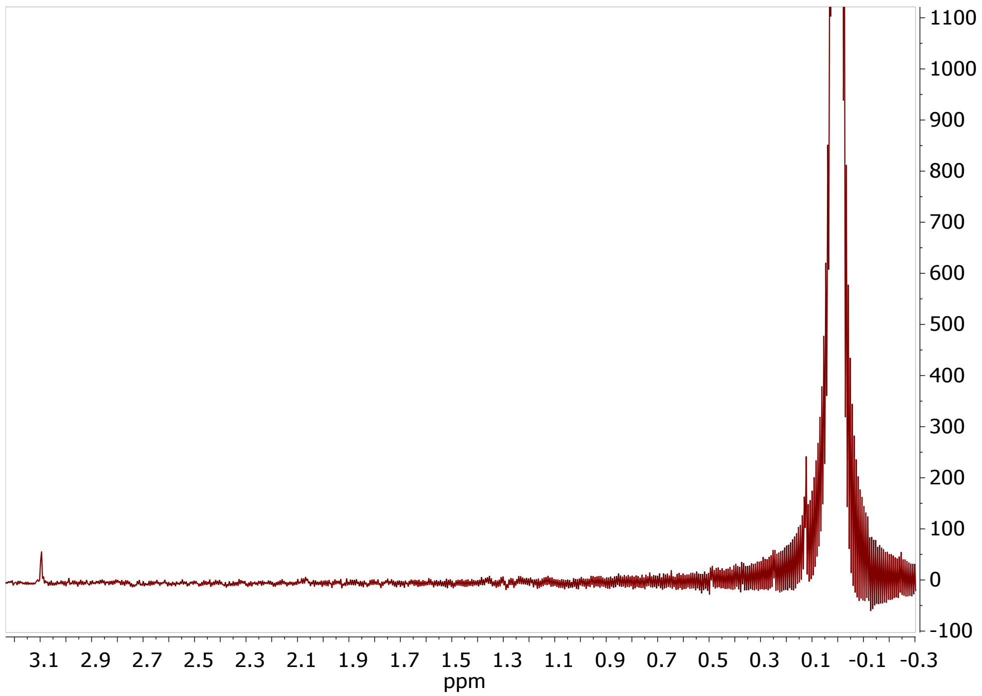

Figure S30. 161 MHz ^{31}P NMR of UiO-66 digested in NaOH D_2O solution. Phosphoric acid was added in a sealed capillary for use as a reference (0 ppm).

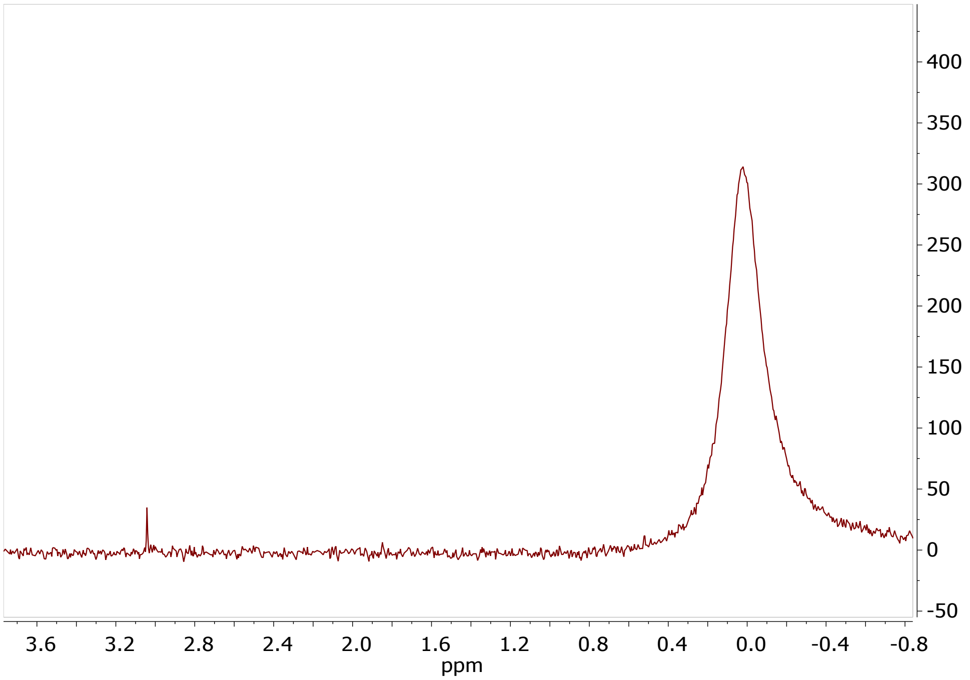

Figure S31. 161 MHz ^{31}P NMR of MOF-808 digested in NaOH D_2O solution. Phosphoric acid was added in a sealed capillary for use as a reference (0 ppm).

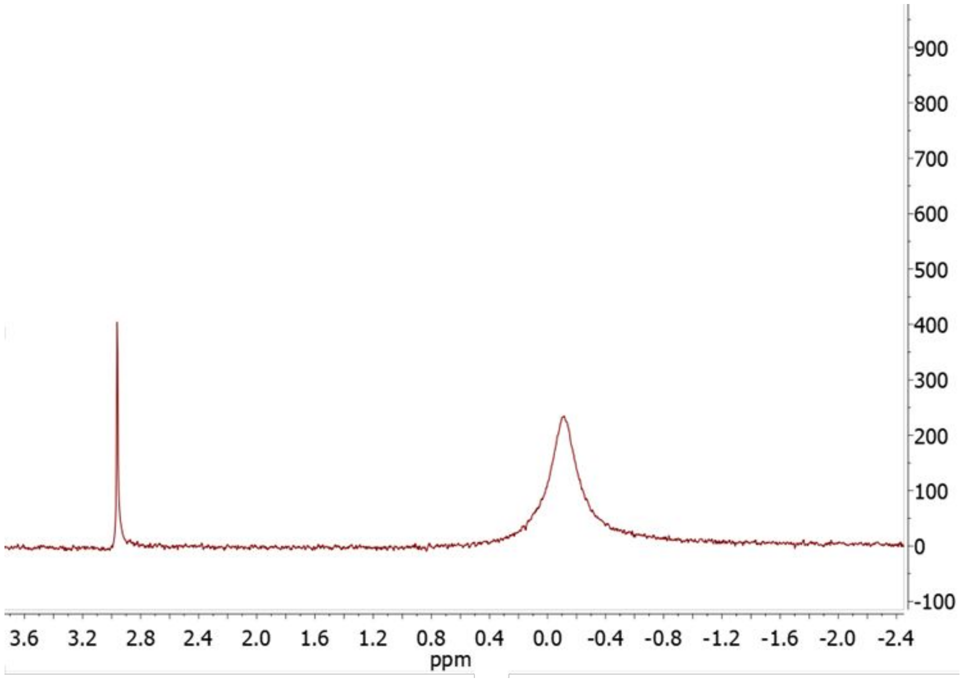

Figure S32. 161 MHz ^{31}P NMR of NU-901 digested in NaOH D_2O solution. Phosphoric acid was added in a sealed capillary for use as a reference (0 ppm).

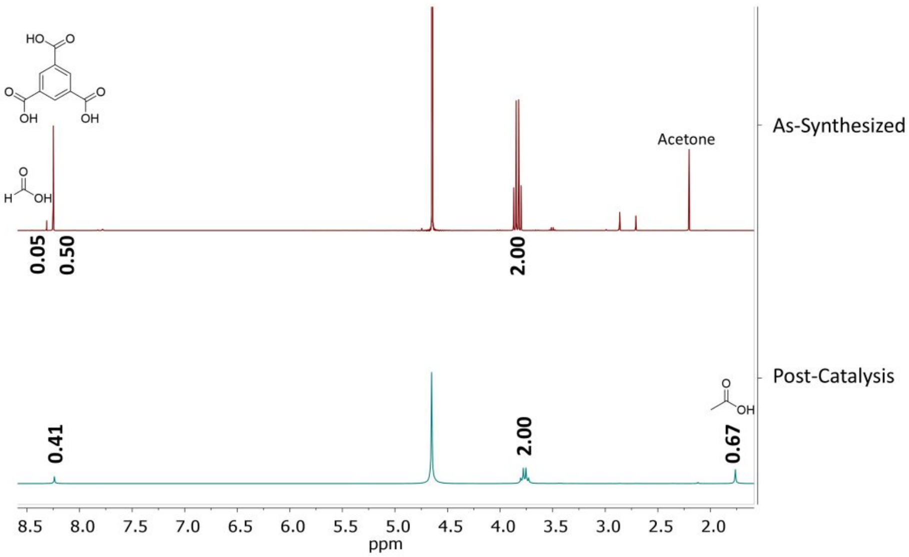

Figure S33. 400 MHz ^{1}H NMR of MOF-808 xerogel before (top) and after (bottom) DMNP hydrolysis experiments digested in NaOH D_2O solution. A standard of 2,2,2-trifluoroethanol was sealed in a capillary tube and added to the NMR tube.

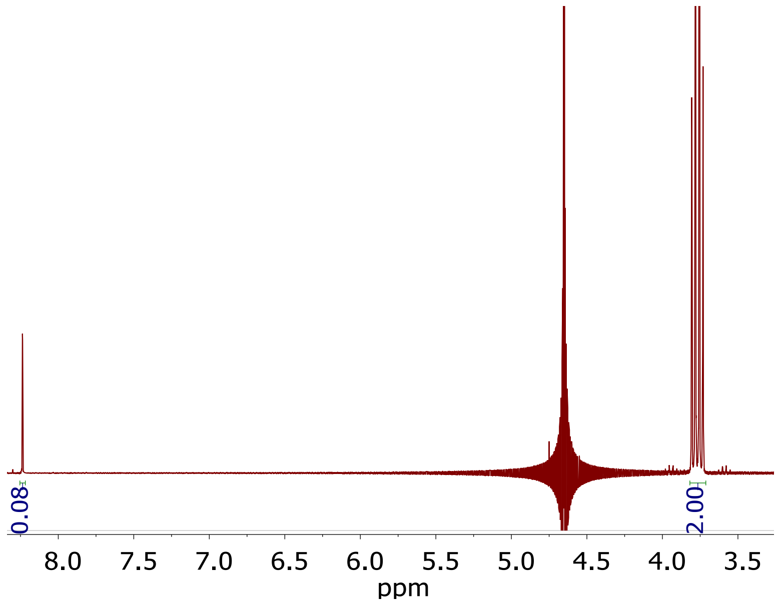

Figure S34. 400 MHz ^{1}H NMR of MOF-808 xerogel after HCl treatment digested in NaOH D_2O solution. A standard of 2,2,2-trifluoroethanol was sealed in a capillary tube and added to the NMR tube.

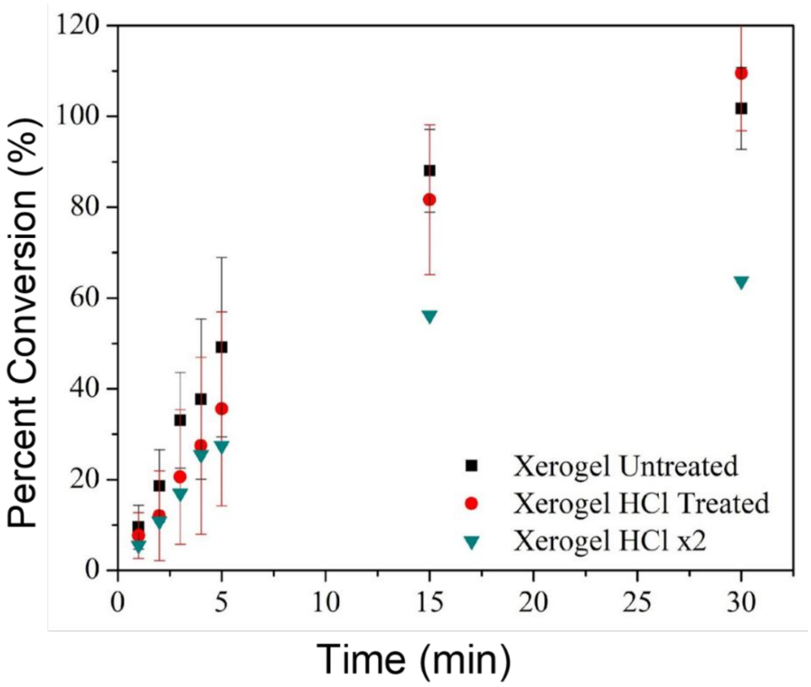

Figure S35. Percent conversion of DMNP based on total hydrolysis product (4-nitrophenol + 4-nitrophenolate) over time for MOF-808 xerogel untreated (black squares), treated once with HCl (red circles), and treated twice with HCl (teal downward triangles).

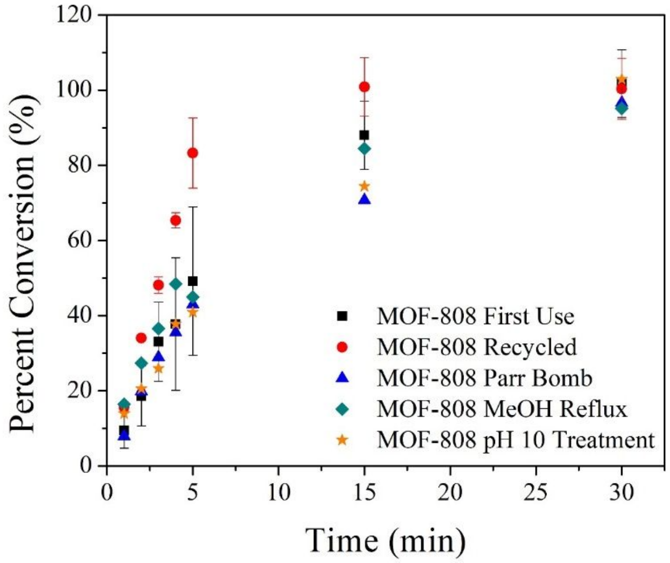

Figure S36. Percent conversion of DMNP based on total hydrolysis product over time for MOF-808 xerogel pretreated with methanol and pH 10 washes.

REFERENCES:

1. Shearer, G. C.; Chavan, S.; Bordiga, S.; Svelle, S.; Olsbye, U.; Lillerud, K. P. Defect Engineering: Tuning the Porosity and Composition of the Metal–Organic Framework UiO-66 via Modulated Synthesis. *Chem. Mater.* **2016,** *28* (11), 3749-3761.

2. Gibbons, B.; Bartlett, E. C.; Cai, M.; Yang, X.; Johnson, E. M.; Morris, A. J. Defect Level and Particle Size Effects on the Hydrolysis of a Chemical Warfare Agent Simulant by UiO-66. *Inorg. Chem.* **2021**, *60*(21), 16378-16387.

3. Mondloch, J. E.; Bury, W.; Fairen-Jimenez, D.; Kwon, S.; DeMarco, E. J.; Weston, M. H.; Sarjeant, A. A.; Nguyen, S. T.; Stair, P. C.; Snurr, R. Q.; Farha, O. K.; Hupp, J. T. Vapor-Phase Metalation by Atomic Layer Deposition in a Metal–Organic Framework. *J. Am. Chem. Soc.* **2013,** *135* (28), 10294-10297.

4. Bueken, B.; Van Velthoven, N.; Willhammar, T.; Stassin, T.; Stassen, I.; Keen, D. A.; Baron, G. V.; Denayer, J. F. M.; Ameloot, R.; Bals, S.; De Vos, D.; Bennett, T. D. Gel-based morphological design of zirconium metal–organic frameworks. *Chem. Sci.* **2017,** *8* (5), 3939-3948.

5. Jiang, J.; Gándara, F.; Zhang, Y.-B.; Na, K.; Yaghi, O. M.; Klemperer, W. G. Superacidity in Sulfated Metal–Organic Framework-808. *J. Am. Chem. Soc*. **2014**, *136*(37), 12844-12847.

6. Garibay, S. J.; Iordanov, I.; Islamoglu, T.; DeCoste, J. B.; Farha, O. K. Synthesis and Functionalization of Phase-Pure NU-901 for Enhanced CO_2 Adsorption: The Influence of a Zirconium Salt and Modulator on the Topology and Phase Purity. *CrystEngComm* **2018**, *20*(44), 7066–7070.
